# Integrator orchestrates RAS/ERK1/2 signaling transcriptional programs

**DOI:** 10.1101/gad.301697.117

**Published:** 2017-09-01

**Authors:** Jingyin Yue, Fan Lai, Felipe Beckedorff, Anda Zhang, Chiara Pastori, Ramin Shiekhattar

**Affiliations:** 1 Department of Human Genetics, Sylvester Comprehensive Cancer Center, University of Miami Miller School of Medicine, Miami, Florida 33136, USA;; 2 Department of Neurological Surgery, Sylvester Comprehensive Cancer Center, University of Miami Miller School of Medicine, Miami, Florida 33136, USA

**Keywords:** Integrator, MAPK signaling pathway, immediate early genes, epidermal growth factor, ERK1/2

## Abstract

Activating mutations in the mitogen-activated protein kinase (MAPK) cascade, also known as the RAS–MEK–extracellular signal-related kinase (ERK1/2) pathway, are an underlying cause of >70% of human cancers. While great strides have been made toward elucidating the cytoplasmic components of MAPK signaling, the key downstream coactivators that coordinate the transcriptional response have not been fully illustrated. Here, we demonstrate that the MAPK transcriptional response in human cells is funneled through Integrator, an RNA polymerase II-associated complex. Integrator depletion diminishes ERK1/2 transcriptional responsiveness and cellular growth in human cancers harboring activating mutations in MAPK signaling. Pharmacological inhibition of the MAPK pathway abrogates the stimulus-dependent recruitment of Integrator at immediate early genes and their enhancers. Following epidermal growth factor (EGF) stimulation, activated ERK1/2 is recruited to immediate early genes and phosphorylates INTS11, the catalytic subunit of Integrator. Importantly, in contrast to the broad effects of Integrator knockdown on MAPK responsiveness, depletion of a number of critical subunits of the coactivator complex Mediator alters only a few MAPK-responsive genes. Finally, human cancers with activating mutations in the MAPK cascade, rendered resistant to targeted therapies, display diminished growth following depletion of Integrator. We propose Integrator as a crucial transcriptional coactivator in MAPK signaling, which could serve as a downstream therapeutic target for cancer treatment.

## Abstract (precis)

In this study, Yue et al. describe a new role for the RNAPII-associated complex Integrator in MAPK signaling. They show that Integrator mediates the transcriptional responsiveness following growth factor signaling, that depletion of Integrator can suppress MAPK signaling to the nucleus, and that Integrator could be targeted in MAPK-driven cancers that are resistant to conventional inhibitors of the MAPK pathway.

The canonical mitogen-activated protein kinase (MAPK) or extracellular signal-related kinase (ERK1/2) cascade is one of the key signaling pathways that transmits growth signals to the nucleus (Chen et al. 1992; Gonzalez et al. 1993; Karin and Hunter 1995). Following its activation, ERK1/2 governs a multitude of transcription factors that regulate expression of genes involved in fundamental cellular processes, including proliferation, differentiation, survival, and motility (Roux and Blenis 2004). Over 150 substrates of ERK1/2 have been identified, and, notably, about half are localized in the nucleus (Yoon and Seger 2006). Perhaps the most studied response following ERK activation is the phosphorylation of transcription factors, including the ETS family members ELK1 and ETS1/2 (Davis et al. 2000; Foulds et al. 2004), that promotes the expression of immediate early genes (IEGs) (Murphy et al. 2002; Foulds et al. 2004; Nelson et al. 2010). Despite the identification of many of these substrates, the precise molecular mechanism by which ERK1/2 activates the expression program of IEGs is strikingly unclear.

Approximately two-thirds of human cancers, including skin, colon, lung, and pancreas; multiple myeloma; and hairy cell leukemia, have aberrations in the ERK1/2 cascade, largely due to activating mutations in signaling intermediates such as EGFR, KRAS, or BRAF (Davies et al. 2002; Garnett and Marais 2004; Dhillon et al. 2007; Bryant et al. 2014). This understanding led to the development of targeted inhibitors against kinase components of the MAPK pathway that could be used for cancer therapy (Roberts and Der 2007; Santarpia et al. 2012). However, the rapid emergence of resistance toward these inhibitors has hindered their therapeutic efficacy (Samatar and Poulikakos 2014). While these cytoplasmic pathways have been the focus of many signaling studies, there is scarcity of knowledge on how such signals are transmitted to the transcriptional machinery beyond that of sequence-specific DNA-binding transcription factors.

We showed previously that the Integrator complex is recruited to the IEGs to coordinate transcriptional initiation and pause release during epidermal growth factor (EGF) stimulation (Gardini et al. 2014). We demonstrated recently that Integrator is also directed to enhancers, where it facilitates transcription of enhancer RNAs (eRNAs) and mediates their 3′ end processing (Lai et al. 2015). The functions of Integrator at IEGs are clearly dictated by growth factor stimulation; however, the signaling pathways that converge on Integrator have yet to be defined. Here, we show that Integrator is a critical downstream node of ERK1/2 signaling in the nucleus. Inhibition of ERK1/2 abrogates the stimulus-dependent recruitment of Integrator and RNA polymerase II (RNAPII) to IEGs and their enhancers. While depletion of Integrator attenuates ERK1/2-mediated transcriptional responsiveness of EGF signaling, knockdown of MED1, MED12, or MED17 subunits of Mediator complex did not alter EGF responsiveness of most genes. Additionally, INTS11 knockdown diminishes the MAPK responsiveness and cellular growth in A375 and A549, cancer cell lines with activating mutations in BRAF and KRAS, respectively. Importantly, depletion of INTS11 diminishes cellular proliferation in A375 cells rendered resistant to MAPK inhibitors, highlighting a possible avenue to overcome drug resistance by targeting INTS11 in cancer cells.

## Results

### Integrator is a key transcriptional coactivator for ERK1/2 signaling

**Figure 1.**
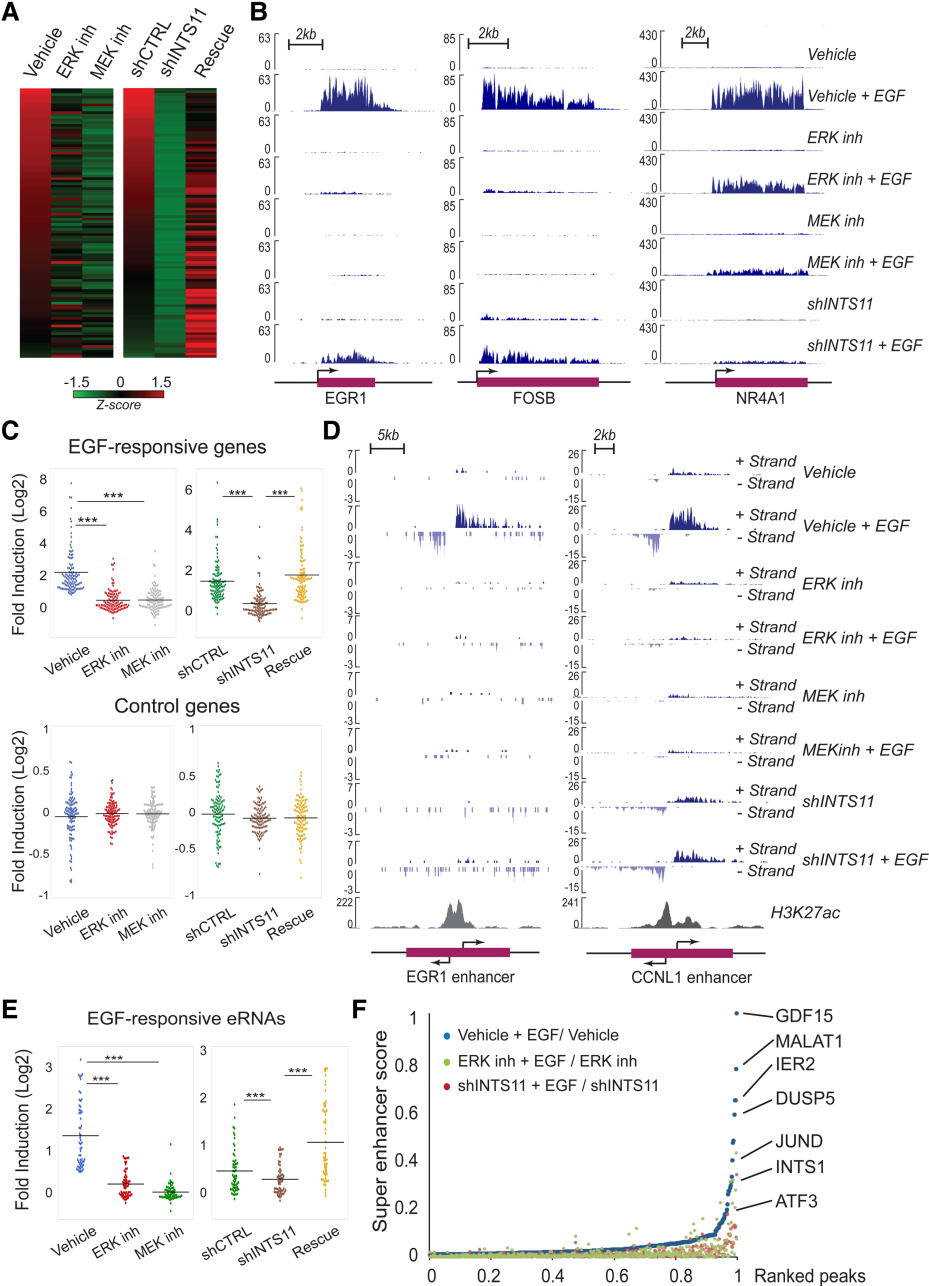
Integrator orchestrates the MAPK-mediated transcriptional response. All genome-wide analyses were performed in at least duplicate biological repeats. (*A*) Heat map representing the fold induction of 106 EGF-induced genes in HeLa cells following treatment with vehicle, ERK inhibitor (SCH772984), MEK inhibitor (PD0325901), or shCTRL, shRNA against INTS11 and INTS11 overexpression (rescue). Each lane represents the fold ratio of gene expression changes before and after 20 min of EGF stimulation. The heat maps are ranked from the highest to the lowest fold induction of EGF-responsive genes. All genes were induced by at least twofold. FPKM (fragments per kilobase per million mapped fragments) > 1; *q*-value < 0.05. *Z*-scores are scaled across rows. The EGF-induced genes are listed in Supplemental Table S1. (*B*) EGF-induced gene expression at *EGR1*, *FOSB*, and *NR4A1* loci were diminished by the presence of ERK inhibitor, MEK inhibitor, or shRNA against INTS11, as revealed by ChromRNA-seq. The *Y*-axis represents the read counts normalized to sequencing depth. (*C*) Dot plots represent significant impairments of EGF responsiveness caused by ERK, MEK inhibition, or INTS11 knockdown. Average expression level of 106 EGF-induced genes or control genes were measured by fold induction after EGF treatment. (***) *P* < 0.001 for all comparisons, *t*-test. (*D*) ERK inhibition or knockdown of INTS11 restrains the activation of EGF-responsive enhancers adjacent to *EGR1* and *CCNL1* gene loci. (*E*) Dot plots indicate similar inhibition of 57 EGF-induced eRNAs by ERK inhibition, MEK inhibition, or INTS11 knockdown. (***) *P* < 0.001 for corresponding comparisons, *t*-test. The EGF-induced eRNAs are listed in Supplemental Table S2. (*F*) The activation of enhancers and superenhancers (SEs) were repressed by ERK inhibition (green) or INTS11 knockdown (red). A full description is in the Materials and Methods. The EGF-induced SEs are listed in Supplemental Table S3.

We showed previously that depletion of Integrator catalytic subunit INTS11 or its largest subunit, INTS1, abrogated EGF transcriptional responsiveness in HeLa cells (Gardini et al. 2014). To dissect the signaling pathway that mediates the EGF transcriptional response of IEGs, we treated HeLa cells with an ERK1/2 (SCH772984) or MEK (PD0325901) inhibitor prior to EGF stimulation and analyzed EGF-responsive gene expression using chromatin RNA sequencing (ChromRNA-seq), which provides for an enriched fraction of nascent RNAs. We found 106 genes that consistently respond (twofold induction; *q*-value < 0.05) to EGF stimulation at the 20-min time point (Supplemental Table S1; Supplemental Fig. S1A). Inhibition of MEK or ERK1/2 impaired activation of most EGF-responsive genes (81 genes were concomitantly inhibited following ERK1/2 or MEK inhibition) (Fig. 1A,B; Supplemental Fig. S1B; Supplemental Table S1). We validated that MEK or ERK1/2 inhibition reduced phosphorylation of RSK1, a downstream target of MAPK signaling. (Supplemental Fig. S2A).

We next compared the diminished transcriptional response incurred by MEK or ERK1/2 inhibition with that following depletion of INTS11. While Integrator knockdown (INTS11 knockdown using an inducible shRNA) did not affect ERK1/2 activation (Supplemental Fig. S2B), it mimicked the pharmacological inhibition of MAPK inhibitors, resulting in the loss of EGF responsiveness in the same group of genes (Fig. 1A–C; Supplemental Fig. S1B). INTS11 depletion resulted in the loss of responsiveness of 77 out of 81 IEGs whose activation was also diminished by inhibition of ERK1/2 and MEK (Supplemental Fig. S1B; Supplemental Table S1). Importantly, ectopic expression of INTS11 from constructs refractory to the action of shRNAs rescued the transcriptional induction of a majority of EGF-responsive genes (70 out of 99 genes were rescued by INTS11 overexpression) (Fig. 1A,C; Supplemental Table S1). Moreover, Integrator depletion and MEK or ERK1/2 inhibition did not affect 106 randomly selected control genes unresponsive to EGF induction (Fig. 1C). Two independent siRNAs against INTS11 or their combination similarly diminished the induction of EGF-responsive genes (Supplemental Fig. S3A). As a complementary approach, either wild type ERK2 or its constitutively active form (R67S and D321N) (Brady et al. 2014) was ectopically expressed in HeLa cells, and their responsiveness to EGF and INTS11 depletion was measured. While ectopic expression of constitutively active *ERK2* increased the basal levels of *NR4A1* and EGR1 expression (compared with that of wild type ERK2), depletion of INTS11 diminished both the basal and EGF-induced expression of *NR4A1* and *EGR1* (Supplemental Fig. S4A–D). These results are consistent with Integrator playing a transcriptional coactivator function in ERK-mediated transcriptional activation.

Next, we analyzed enhancer activation by measuring the response of EGF-stimulated eRNAs at enhancers and superenhancers (SEs). MEK or ERK1/2 inhibition or INTS11 knockdown diminished the EGF-induced eRNA induction at enhancers and SEs, similar to that of protein-coding genes (Fig. 1D–F; Supplemental Fig. S2C; Supplemental Tables S2, S3; data not shown). These results demonstrate that Integrator functions as a critical coactivator of ERK1/2-responsive IEGs within the initial wave of transcriptional activation.

### Integrator is recruited to chromatin by activated ERK1/2

**Figure 2.**
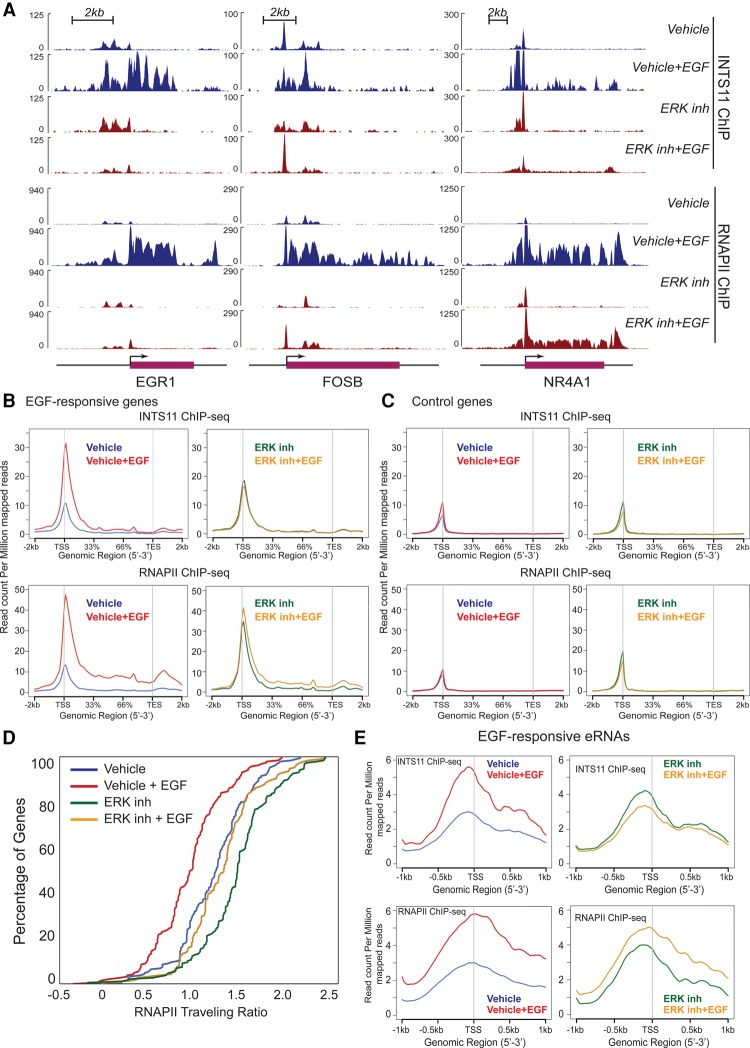
Pharmacological inhibition of the MAPK pathway diminishes stimulus-induced Integrator recruitment. All experiments were performed in at least two biological replicates. (*A*) The presence of ERK inhibitor (SCH772984) affects the dynamics of INTS11 and RNAPII recruitment at *EGR1*, *FOSB*, and *NR4A1* loci. Diagrams of the *EGR1*, *FOSB*, and *NR4A1* genomic regions are at the *bottom*. (*B*,*C*) Average profiles of INTS11 (*top*) and RNAPII (*bottom*) recruitment at 106 EGF-induced genes (*B*) and 106 control genes that were randomly selected (see the Materials and Methods for more details). (*C*). ChIP-seq was performed before and after 20 min of EGF induction with or without ERK inhibitor treatment. (*D*) The RNAPII traveling ratio at 106 EGF-induced gene loci was measured. The ratio was calculated as log_10_ of read density at the transcription start site (TSS)/read density over the gene body. All distributions were significantly different. *P* < 0.001, Kolmogorov-Smirnov test. (*E*) Average profiles of INTS11 or RNAPII recruitment at 57 EGF-induced enhancers (see the Materials and Methods).

We next asked whether ERK1/2 signaling drives Integrator recruitment following EGF stimulation. We performed ChIP-seq (chromatin immunoprecipitation [ChIP] combined with high-throughput sequencing) of INTS11 and RNAPII before and after the treatment of cells with ERK1/2 inhibitor (SCH772984). Inhibition of ERK1/2 signaling diminished the immediate–early recruitment of Integrator and RNAPII to EGF-responsive IEGs (Fig. 2A,B). This was manifested by decreased occupancy of Integrator and RNAPII at the 5′ end and body of EGF-responsive genes (Fig. 2A–C). In agreement with our previously reported effects of INTS11 depletion (Gardini et al. 2014), analysis of the RNAPII traveling ratio indicated that ERK1/2 inhibition substantially decreased transcriptional elongation following EGF induction (Fig. 2D). In addition, treatment of serum-starved cells with ERK1/2 inhibitor prior to EGF stimulation similarly resulted in increased pausing of RNAPII, as reflected by the accumulation of RNAPII at the 5′ end of EGF-responsive genes and, to a lesser extent, at the control gene set (Fig. 2B,C). There was no change in transcriptional activity of either gene set following treatment of starved cells with ERK1/2 inhibitor (Supplemental Table S4), consistent with the increased residence of nonproductive paused RNAPII at the 5′ ends of both gene sets. Finally, consistent with Integrator's role in eRNA production, ERK1/2 inhibition abrogated the recruitment of Integrator and RNAPII to EGF-induced enhancers (Fig. 2E). These results demonstrate that ERK1/2 signaling funnels through the Integrator complex and promotes its recruitment to IEGs. The impaired transcriptional response that follows INTS11 knockdown indicates that Integrator is a critical downstream component of MAPK signaling in the nucleus.

### Integrator fulfills a specific coactivation function in MAPK signaling

**Figure 3.**
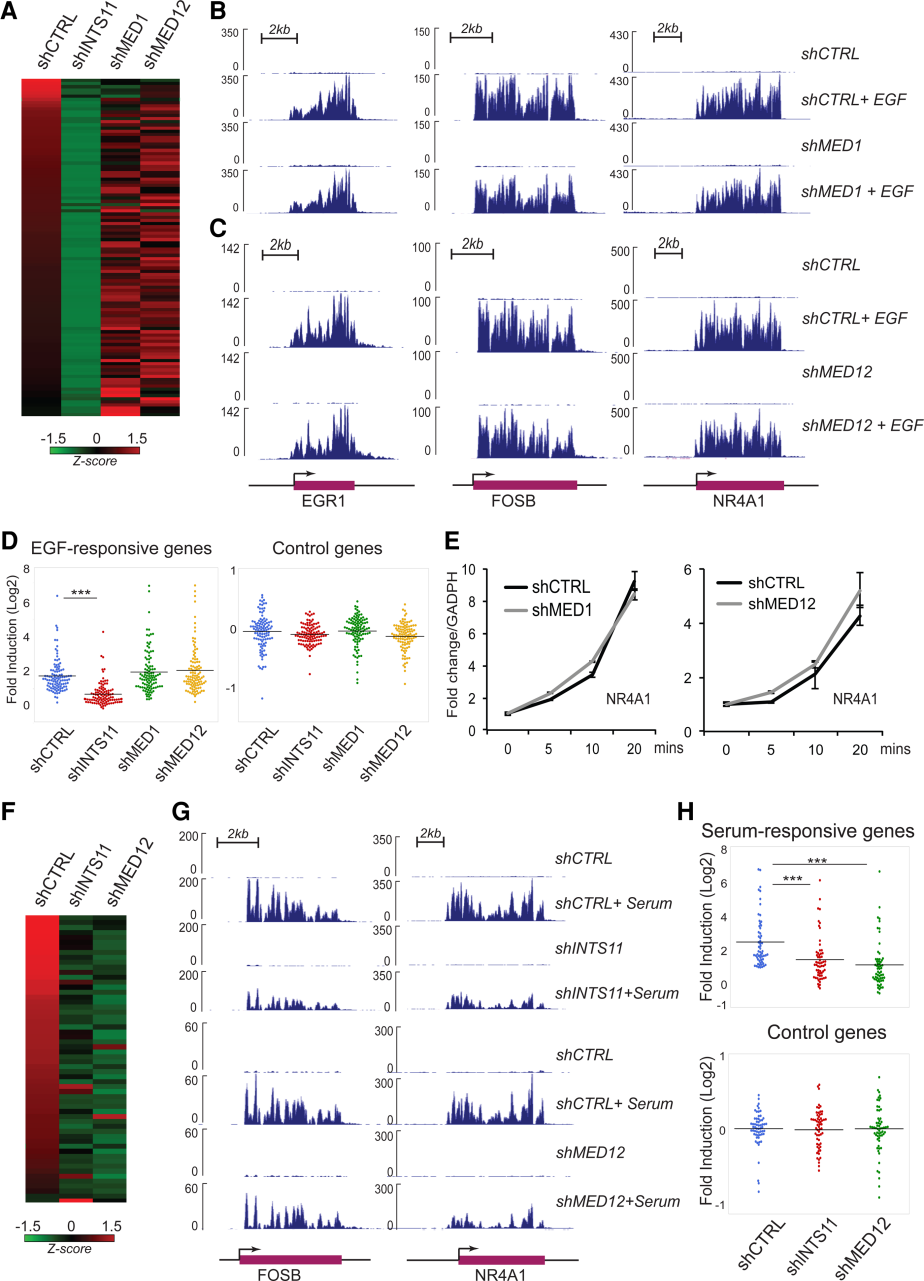
Depletion of Mediator subunits does not significantly affect MAPK responsiveness. (*A*) Heat map representing the fold induction of 106 EGF-induced genes in HeLa cells following treatment with shCTRL or shRNA against INTS11, MED1, or MED12. All experiments were performed in at least two biological repeats. Each lane represents the fold ratio of gene expression changes before and after 20 min of EGF stimulation. The heat map is ranked from the highest to the lowest fold induction of EGF-responsive genes. (*B*,*C*) EGF-induced gene expression at *EGR1*, *FOSB*, and *NR4A1* loci did not change by the presence of shRNA control or shRNA against MED1 (*B*) or MED12 (*C*), as revealed by ChromRNA-seq. (*D*) Dot plots represent significant impairments of EGF-induced activation by INTS11 knockdown but not by knockdown of MED1 or MED12. Average expression levels of 106 EGF-induced genes or control genes were measured by fold induction after EGF treatment. (***) *P* < 0.001 for all comparisons, *t*-test. (*E*) Knockdown of MED1 or MED12 does not affect EGF-induced *NR4A1* gene activation. The gene transcription level was measured before and after 5, 10, and 20 min of EGF induction using quantitative RT–PCR (qRT–PCR). Shown in the figure is the average from three independent experiments. Error bars represent SEM. (*F*) Heat map representing the fold induction of 60 serum-induced genes after treatment with shCTRL or shRNA against INTS11 or MED12. Each lane represents the fold ratio of gene expression changes before and after 20 min of serum stimulation. All genes were induced by at least twofold. FPKM > 1; *q*-value < 0.05. The heat map is ranked from the highest to the lowest fold induction of serum-responsive genes. (*G*) Serum-induced gene expression at *FOSB* and *NR4A1* loci was reduced by the presence of shRNA against INTS11 or MED12. (*H*, *top*) Dot plots showing that loss of INTS11 or MED12 abrogates the serum-induced transcriptional activation on serum-responsive genes. (*Bottom*) There is no significant change in control genes. The serum-responsive genes are listed in Supplemental Table S5. (***) *P* < 0.001, *t*-test.

In multicellular organisms, RNAPII associates with two major multiprotein complexes: the Mediator complex, which is conserved in unicellular eukaryotes, and Integrator, which evolved later following the unicellular-to-multicellular transition. We depleted two subunits of Mediator complex, MED1 and MED12, and assessed their roles in EGF responsiveness genome-wide. In contrast to INTS11 depletion, knockdown of MED1 or MED12 did not significantly alter the EGF-mediated induction of IEGs (Fig. 3A–D; Supplemental Figs. S5A, S6A, B). We found that 16 of 81 MAPK-responsive genes decrease their transcriptional induction following depletion of MED1 or MED12 (Supplemental Fig. S6B; Supplemental Table 1). This overall lack of responsiveness is also reflected in a time course for EGF-induced transcriptional stimulation following depletion of MED1, MED12, or MED17 (Fig. 3E; Supplemental Fig. S5B). Despite the lack of change in most EGF-induced genes following depletion of MED1, MED12, or MED17, we detected ERK1/2-depedent recruitment of MED1 and MED12 following EGF stimulation (Supplemental Fig. S7A–D). Since Mediator is composed of >20 subunits, it is plausible that an unexamined component of the Mediator complex contributes to the MAPK response of additional genes.

Importantly, consistent with previous reports (Donner et al. 2010), depletion of MED12 significantly attenuated transcriptional activation following serum stimulation, which contains a milieu of growth factors, including EGF (Fig. 3F–H; Supplemental Table S5). Depletion of INTS11 similarly attenuated the serum response, indicating that while Integrator has a broad role in growth factor signaling, Mediator subunits may exert their function on a specific cytokine signaling pathway.

### Activated ERK1/2 is recruited to the promoters of IEGs and phosphorylates INTS11

**Figure 4.**
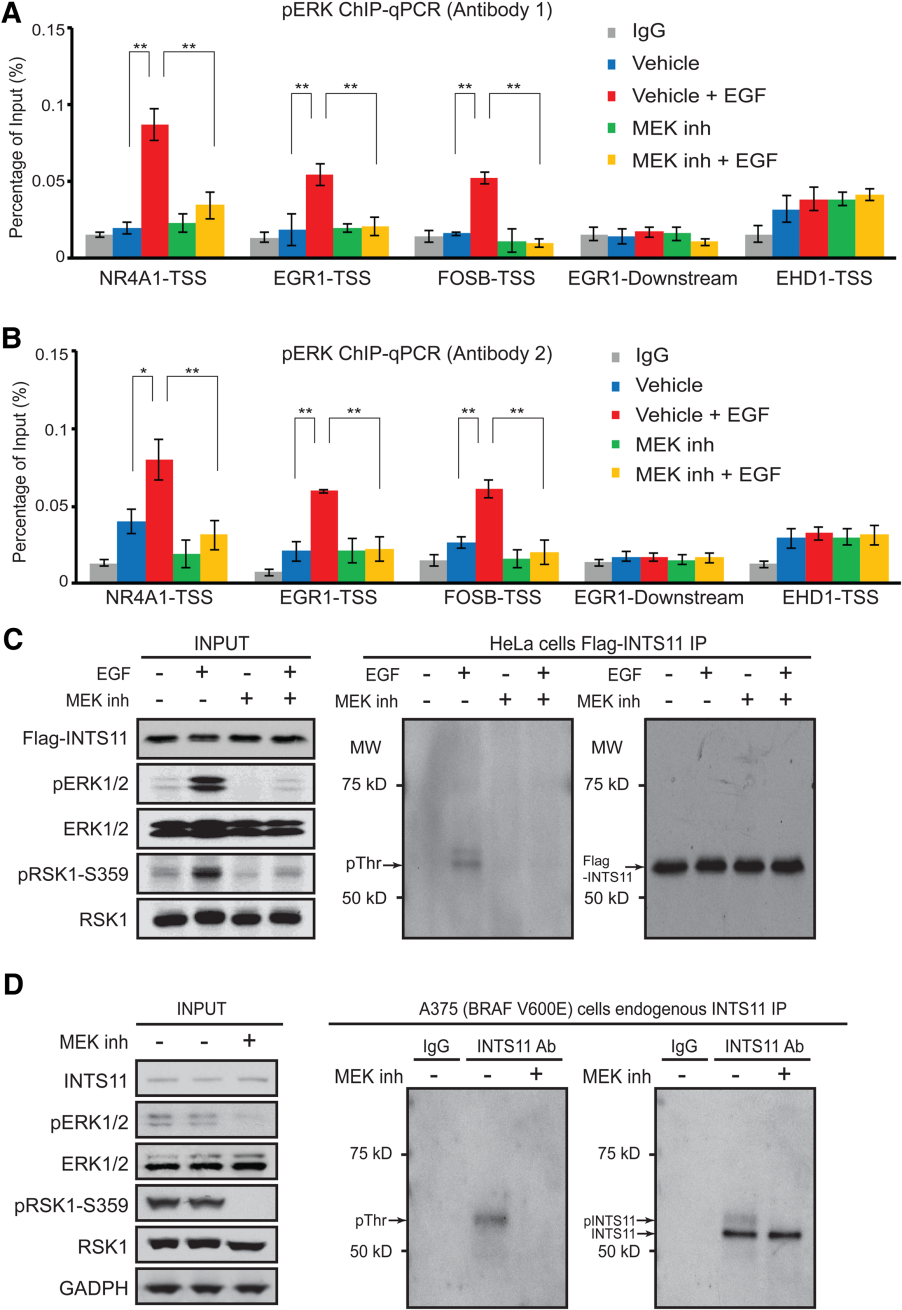
Integrator is phosphorylated following MAPK induction, and phoshpho-ERK1/2 is recruited to EGF-responsive genes upon pathway activation. (*A*,*B*) Phospho-ERK1/2 ChIP-qPCR (ChIP combined with qPCR) with antibody-1 (Cell Signaling Technology, no. 4370) and antibody-2 (Invitrogen, no. 700012). The cells were collected before and after 20 min of EGF induction with or without the presence of MEK inhibitor (PD0325901). After ChIP, qPCR was performed with primers located in the TSS region of the *NR4A1*, *EGR1*, and *FOSB* genes. The primer pairs located in the TSS region of the *EHD1* gene and ∼3 kb downstream from the transcription end site (TES) of the *EGR1* gene were used as negative controls. The average from at least three independent experiments is shown. (*) *P* < 0.05; (**) *P* < 0.01, *t*-test. (*C*,*D*) Western blots of immunoprecipitation for exogenous expression of Flag-INTS11 in HeLa cells (*C*) and endogenous INTS11 in A375 (BRAF_V600E) cells (*D*). Phosphorylated INTS11 protein was detected by specific antibody against phosphothreonine (Cell Signaling, no. 9381).

To further examine the mechanism by which ERK1/2 signaling leads to the activation of IEGs, we asked whether active ERK1/2 is recruited to *NR4A1*, *EGR1*, and *FOSB* in a signal-dependent manner. We used two different antibodies directed to phosphorylated ERK1/2 (pERK) to assess the chromatin residence of pERK. We observed a robust and specific EGF-induced recruitment of pERK to the transcription start sites (TSSs) of *NR4A1*, *FOSB*, and *EGR1* that diminished following inhibition of MEK (Fig. 4A,B). These results indicate that pERK is recruited to the promoters of IEGs following activation of the MAPK cascade.

To ask whether pERK can directly phosphorylate INTS11, we isolated Integrator from a HeLa cell line stably expressing Flag-INTS11 and assessed phosphorylation of INTS11 following EGF stimulation. While we did not observe a signal corresponding to phosphoserine (data not shown), we detected a phosphothreonine signal in a molecular mass range close to Flag-INTS11 on an SDS-PAGE following EGF stimulation that is lost after inhibition of MEK (Fig. 4C). To confirm that this signal corresponds to INTS11, we immunoprecipitated the endogenous protein using antibodies against INTS11 from A375 cells with activated BRAF and measured the extent of phosphorylation using phosphotheronine antibodies. We detected two bands corresponding to INTS11 following Western blot analysis using INTS11 antibodies (Fig. 4D). Moreover, we found a phosphothreonine signal at a molecular mass similar to phosphorylated INTS11 (Fig. 4D). Importantly, treatment of A375 cells with MEK inhibitor resulted in loss of phosphorylation signal concomitant with the loss of the higher-molecular-mass band corresponding to INTS11 (Fig. 4D). Taken together, these results are consistent with phosphorylation of INTS11 by pERK following EGF signaling.

### INTS11 knockdown diminishes ERK1/2 responsiveness in cancers with activated MAPK

**Figure 5.**
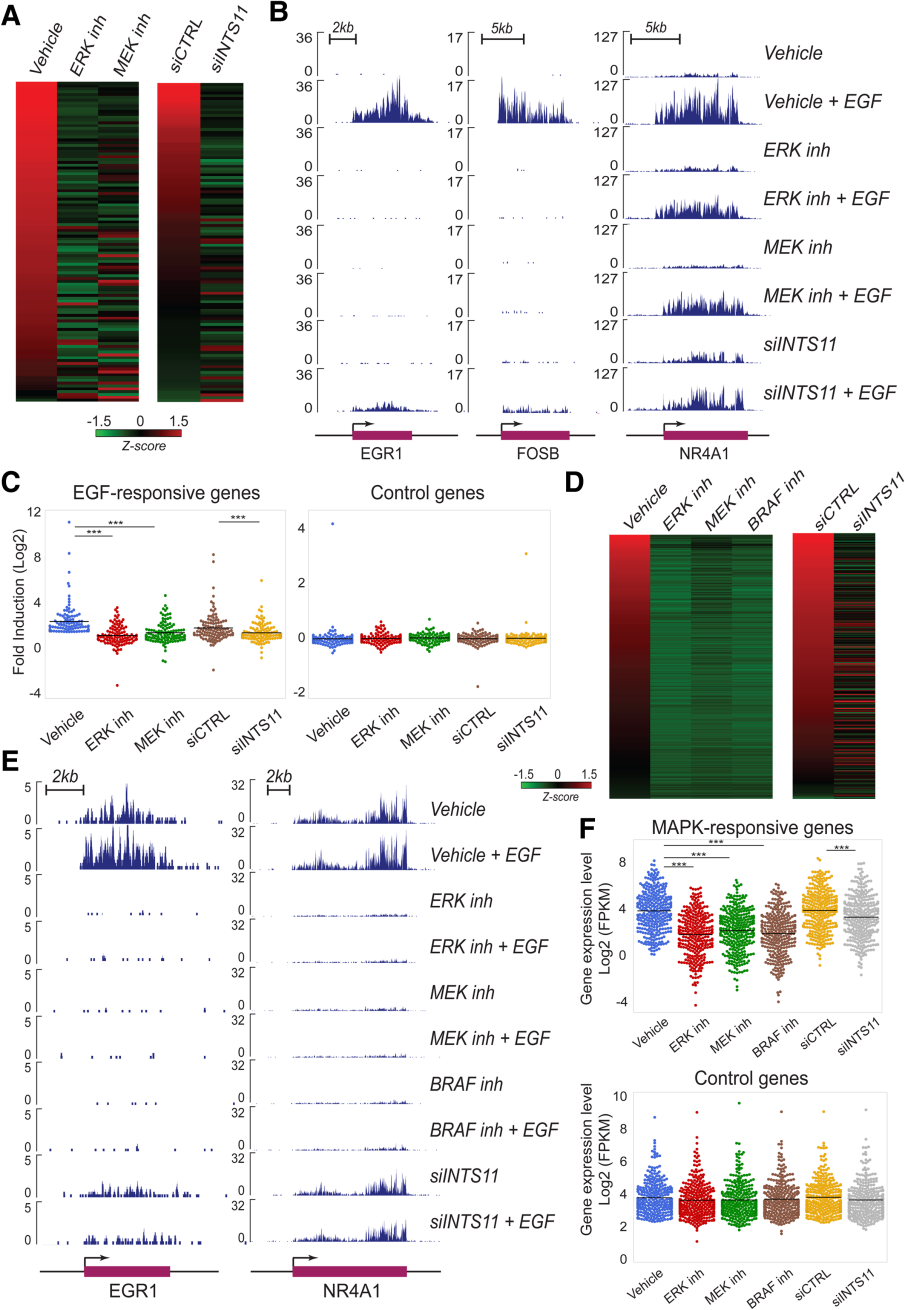
Integrator directs MAPK transcriptional responsiveness in cancers with MAPK-activating mutations. All experiments were performed in at least two biological repeats. (*A*–*C*) Lung adenocarcinoma cells (A549) with KRAS-activating mutation. (*D*–*F*) Melanoma cells with the V600E BRAF mutation (A375). Heat maps representing the activation of EGF-responsive genes in A549 cells (*A*) or genes responsive to MAPK inhibitors in A375 cells (*D*) treated with DMSO, ERK inhibitor (SCH772984), MEK inhibitor (PD0325901), BRAF inhibitor (vemurafenib; only in *D*), nontargeting siRNA, or siRNA against INTS11. Heat maps are ranked from the highest to the lowest fold induction of EGF-responsive genes (*A*) or highest FPKM (*D*). The EGF-responsive genes in A549 cells are listed in Supplemental Table S6, and the genes responsive to MAPK inhibitors in A375 cells are listed in Supplemental Table S7. (*B*,*E*) ChromRNA-seq analysis of EGF-induced gene expression at *EGR1*, *FOSB*, and *NR4A1* loci restrained by ERK1/2 inhibition, MEK inhibition, BRAF inhibition (only in *E*), or siRNA against INTS11. (*C*,*F*) Dot plots represent the fold induction of EGF-responsive genes (*C*) and gene expression level of MAPK-responsive genes (*F*) after treatment with inhibitors or siRNAs. (***) *P* < 0.001 for corresponding comparisons, *t*-test. All EGF genes were induced by at least twofold (FPKM > 1; *q*-value < 0.05), and the genes responsive to MAPK inhibitors were reduced by at least twofold (FPKM > 1; *q*-value < 0.05) in the three treatments (MEK, ERK, and BRAF inhibitors).

We next asked whether INTS11 knockdown affects the MAPK-mediated responsiveness in cancer cell lines with activating mutations in the MAPK signaling pathway. We treated A549 lung adenocarcinoma cells containing mutations in KRAS (homozygous G12S mutation) with either ERK1/2 or MEK inhibitors (SCH772984 and PD0325901, respectively) prior to stimulation with EGF, similar to the protocols that we used for HeLa cells (Supplemental Fig. S8A). Treatment of A549 with either inhibitor specifically diminished the EGF responsiveness of most EGF-responsive genes (99 out of 112 EGF-responsive genes diminished their responsiveness following treatment with either ERK1/2 or MEK inhibitor) (Fig. 5A–C; Supplemental Table S6; Supplemental Fig. S9A,B). Depletion of Integrator displayed a loss of transcriptional induction following EGF stimulation similar to that observed following treatment with MAPK pathway inhibitors (78 out of 112 EGF-responsive genes were concomitantly inhibited by INTS11 depletion or treatment with MAPK inhibitors) (Fig. 5A–C; Supplemental Figs. S3B, S9B).

We extended our analyses to A375 melanoma cells, which contain an activating V600E mutation in BRAF. We treated A375 cells with inhibitors targeting mutant BRAF, MEK, and ERK1/2 to arrive at a set of hyperactivated MAPK-responsive genes (299 genes) that significantly diminished their transcription (reduction by twofold; *q*-value < 0.05) upon treatment with the three inhibitors (Fig. 5D–F; Supplemental Table S7; Supplemental Figs. S8B, S9C). Interestingly, the V600E mutation in BRAF rendered these cells nearly unresponsive to EGF stimulation (Fig. 5E). Importantly, depletion of INTS11 resulted in a significant cessation of MAPK-responsive transcriptional activation in genes that responded to MAPK pathway inhibitors (117 of 299 MAPK-responsive genes diminished their expression) (Fig. 5D–F; Supplemental Figs. S3C, S9D). This was specific, as 299 randomly chosen control genes were unaffected following treatment with MAPK pathway inhibitors or INTS11 knockdown (Fig. 5F). A375 cells responded similarly to MAPK pathway inhibition or Integrator depletion regardless of EGF stimulation (Fig. 5E; Supplemental Fig. S8C,D). Overall, BRAF-activated cells displayed a greater inhibition of MAPK-responsive gene expression following treatment with MAPK pathway inhibitors compared with that after Integrator depletion (Fig. 5D; Supplemental Fig. S9D). This most likely reflects the activation of a large number of immediate–late genes due to the constitutive activation of the MAPK pathway in A375 cells. Nevertheless, these results demonstrate that Integrator could be used as a target in cancer cells with activating mutations in the MAPK pathway to decrease ERK1/2-mediated transcriptional induction.

### Knockdown of INTS11 inhibits proliferation of cancers with activated MAPK

**Figure 6.**
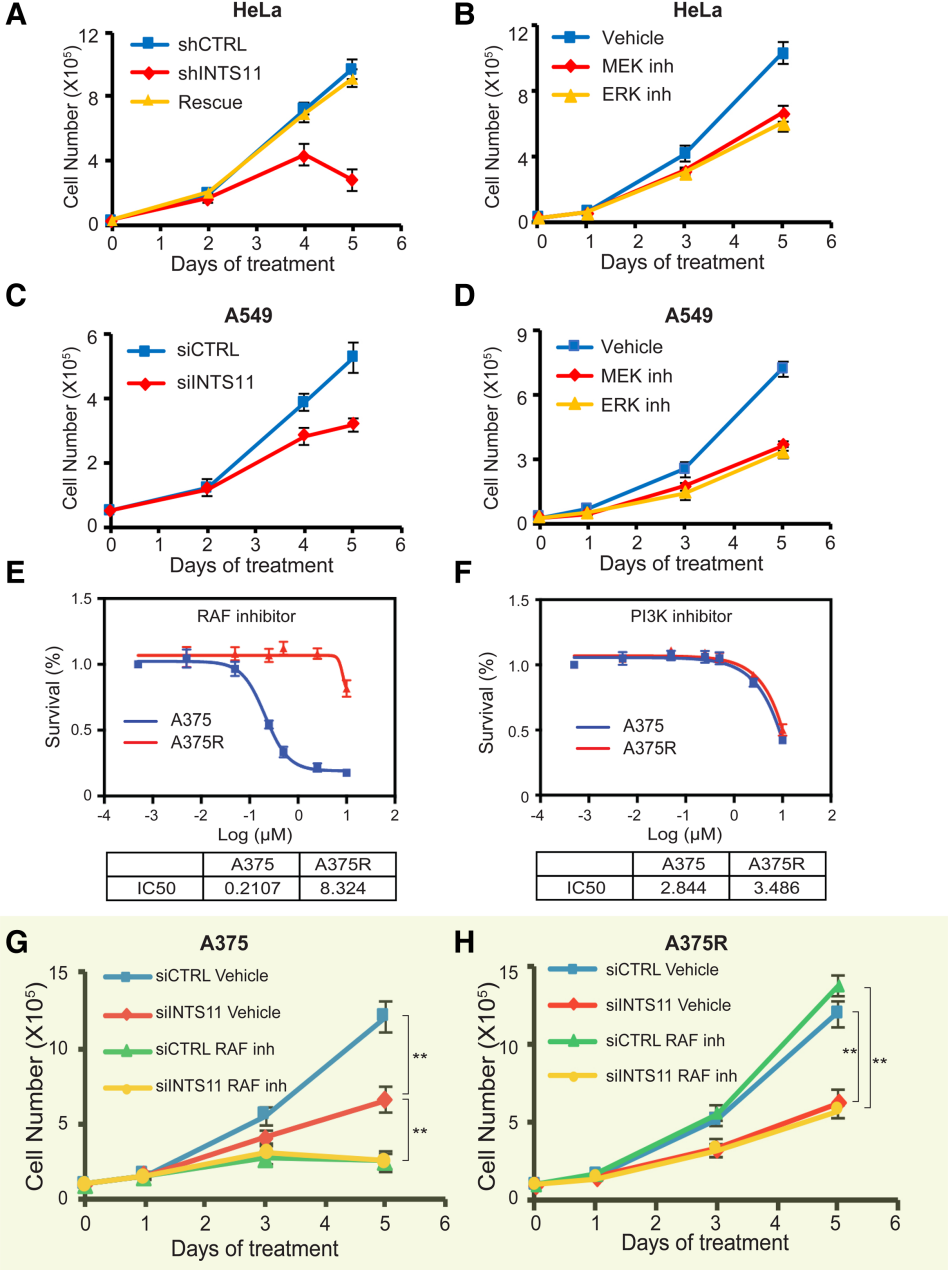
Integrator targeting inhibits growth of cancer cells with MAPK mutations. (*A*–*D*) Cell growth curves for HeLa (*A*,*B*) or KRAS mutant lung cancer cell A549 (*C*,*D*) depleted of INTS11 or treated with MAPK inhibitors. (*A*) This growth defect was fully rescued by restoring the expression of INTS11 (rescue). (*B*) Blocking MAPK signaling by treating cells with MEK inhibitor (0.5 µM PD0325901) or ERK inhibitor (1 µM SCH772984) resulted in cell growth inhibition. (*C*,*D*) In A549 cells, depletion of INTS11(*C*) or inhibition of MAPK signaling (*D*) impaired cell growth. (*E*,*F*) IC50s (concentrations of 50% growth inhibition) of RAF inhibitor (vemurafenib; *E*) and PI3K inhibitor (LY294002; *F*) for BRAF V600E mutant melanoma cell A375 (*E*) and RAF inhibitor-resistant line A375R (*F*) (see the Materials and Methods for details). (*G*,*H*) Cell growth curves for A375 (*G*) and A375R (*H*) cells. The cells were transfected with nontargeting siRNA (siCTRL) or siRNA against INTS11 (siINTS11) on day 0. Six hours after transfection, the cells were fed with medium containing vehicle or RAF inhibitor (0.4 µM vemurafenib) for up to 5 d. siRNA transfection was repeated on day 2. The average of three independent experiments is shown. (**) *P* < 0.01.

We compared the effectiveness of MEK and ERK1/2 inhibitors with INTS11 knockdown for cellular growth suppression in multiple cancer cell lines (Supplemental Fig. S10). INTS11 depletion leads to a specific decrease in cellular growth in HeLa cells, which is completely reversed by ectopic expression of INTS11 refractory to the action of shRNA (Fig. 6A). Treatment of HeLa cells with inhibitors of MEK or ERK1/2 kinases displayed a similar decrease in cellular growth (Fig. 6B). We next measured cellular growth in KRAS mutant A549 cells following knockdown of INTS11 or MAPK inhibition (Supplemental Fig. S10A,B). Both treatments resulted in a significant reduction of cellular growth (Fig. 6C,D).

Next, we compared the loss of INTS11 and inhibition of the MAPK pathway in A375 cells (Supplemental Fig. S10A,B). We also assessed cellular growth following a similar treatment in A375 cells rendered resistant to vemurafenib (A375R), the specific inhibitor of V600E mutations of BRAF. Despite their resistance to BRAF inhibition, A375R cells displayed sensitivity to inhibition of PI3 kinases (Fig. 6E,F). Importantly, while A375 and A375R responded equally to INTS11 depletion, concomitant treatment of these cells with vemurafenib following INTS11 depletion did not result in further suppression of growth (Fig. 6G,H). These results are consistent with the conclusion that BRAF and INTS11 participate in the same signaling cascade and further highlight targeting of INTS11 as a possible therapeutic opportunity in the treatment of human melanoma refractory to BRAF inhibition. Taken together, these results support the notion that cancer cell lines with activating mutations in MAPK signaling are sensitive to Integrator perturbations.

## Discussion

Genetic aberrations in components of the MAPK cascade underlie some of the deadliest human cancers, including those found in the lung and pancreas. Despite tremendous advances toward understanding the molecular basis of MAPK signaling in the cytoplasm, our knowledge of how the activation of ERK1/2, the last cytoplasmic component of the pathway, is translated into a rapid and coordinated transcriptional response in the nucleus is sorely lacking. Although it is well known that ERK1/2 phosphorylates a set of transcription factors, predominantly the ETS-related family members, the precise molecular mechanisms that lead to transcriptional induction have not been elucidated. Previous studies have implicated the transcriptional coactivators CBP/p300 or components of the Mediator complex in MAPK signaling (Janknecht and Nordheim 1996; Pandey et al. 2005; Wang et al. 2005; Jun et al. 2010; Galbraith et al. 2013). However, these studies are generally limited to the analysis of a single or a small number of MAPK-responsive genes in a specific cell line (Jun et al. 2010; Galbraith et al. 2013). Here, we demonstrate that Integrator confers the ERK1/2 transcriptional induction to a large repertoire of MAPK-responsive genes in multiple cancer cell lines, including those with cancer-causing activating mutations in components of MAPK signaling.

**Figure 7.**
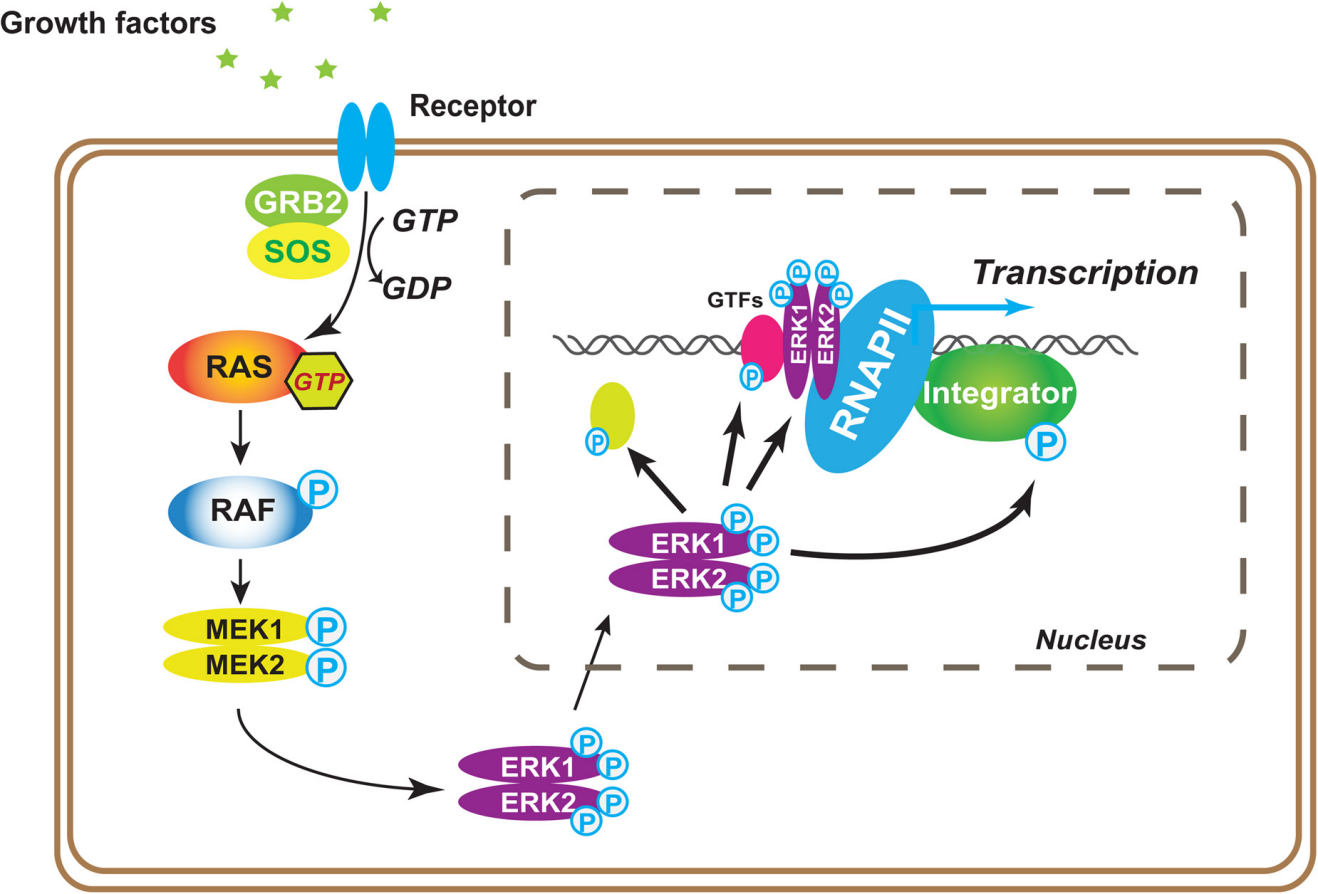
Model depicting Integrator's role as a critical node in MAPK transcriptional induction. Following growth factor stimulation, ERK1/2 are phosphorylated at Tyr204/187 and then Thr202/185 through the canonical RAS–RAF–MEK signaling cascade. The phosphorylation of both tyrosine and threonine enables the enzyme activation. Activated ERK1/2 translocate into the nucleus and regulate the IEG response through phosphorylating related nuclear transcription factors. Integrator is phosphorylated and recruited onto chromatin after the activation of MAPK signaling, which is required for growth factor-stimulated gene activation.

Our results point to the emergence of Integrator as a critical node in the transcriptional response downstream from growth factor signaling (Fig. 7). We show that inhibition of the MAPK cascade abrogates the stimulus-dependent recruitment of Integrator. There are multiple mechanisms by which Integrator could be recruited to pERK1/2-responsive genes. Phosphorylation of a specific transcription factor and/or RNAPII by ERK1/2 could result in increased recruitment of RNAPII and Integrator to MAPK-responsive genes (Rowan et al. 2000). Concomitantly, subunits of Integrator could be the direct target of MAPK signaling. Indeed, we show that INTS11 is phosphorylated by ERK1/2, and it is likely that other subunits of the Integrator complex could be the target of the MAPK and other signaling pathways known to regulate gene expression.

There has been evidence showing the association of ERK1/2 with chromatin in mouse stem cells (Tee et al. 2014). Importantly, we show that activated ERK1/2 is recruited to the promoter of IEGs in a signal-dependent manner. Therefore, it is likely that following activation of the MAPK pathway, ERK1/2 functionally associates with Integrator and perhaps other components of the transcriptional machinery at MAPK-responsive genes.

It is interesting that while both Mediator and Integrator complexes play a transcriptional coactivation function in response to serum stimulation, only Integrator plays a broad role in EGF-induced MAPK signaling. Serum contains a number of growth factors in addition to EGF. Therefore, it is likely that Mediator functions in the transcriptional responsiveness of important cytokine signaling pathways that need to be further elucidated. Moreover, since Mediator contains >30 subunits, it is possible that one or more subunits not examined in our study may be important in MAPK signaling. Single-cell eukaryotic organisms such as yeast do not possess Integrator. Therefore, it is likely that during evolution, upon increasing complexity of the genome, Integrator was designated with specific coactivating functions that, in simpler systems, were performed by the Mediator complex.

We placed the Integrator complex as a critical component of the MAPK pathway necessary for the induction of IEGs. Targeting components of the MAPK pathway in cancers with activating mutations using kinase inhibitors have been plagued with the rapid emergence of resistance. The identification of Integrator, especially its catalytic subunit, INTS11, as a critical downstream component of this pathway opens the way to the development of small molecule inhibitors to Integrator, which may overcome the present therapeutic difficulties in targeting the MAPK pathway in cancer.

## Materials and methods

### RNA-seq and CHIP-seq

RNA-seq and ChIP-seq were performed as described previously (Gardini et al. 2014; Lai et al. 2015). Briefly, NEBNext Ultra RNA and the ChIP-seq library preparation kit for Illumina (New England Biolabs, E7420 and E6240) were used to prepare the sequencing library. Sequencing was performed as a 75-base-pair (bp) single-end run using the NextSeq 500 high-output kit provided by the Oncogenomics Core Facility at the Sylvester Comprehensive Cancer Center at the University of Miami Miller School of Medicine.

### RNA-seq analysis

RNA-seq data were aligned to human genome (hg19 version) using TopHat2 (Kim et al. 2013), and differential expression analysis was performed using Cuffdiff 2.2.1 (Trapnell et al. 2010) with default parameters. The genes with differential expression (EGF-responsive genes) were considered significant when the *q*-value was <0.05, fold change was >2, and FPKM (fragments per kilobase per million mapped fragments) was >1 for protein-coding genes and when FPKM was >0.5 (HeLa: 106 genes, A549: 112 genes) and fold change was >1.6 for eRNAs. Genes responsive to MAPK inhibitors were defined as the common differentially expressed genes in the treatments with ERK, MEK, and BRAF inhibitors with *q*-value <0.05, fold change >2, and FPKM >1 (A375: 299 genes). Control genes were randomly selected from the group of genes that were not differentially expressed in all conditions (*q*-value > 0.05, fold change < 2 or fold change > −2, and FPKM > 1). To compare the genes affected by each condition, we first checked whether there was at least a 50% reduction in the top 30% of EGF-responsive genes (HeLa or A549 cells) by fold induction, and, for the remaining 70% of genes, we followed the criteria of *q*-value > 0.05 and fold change < 2 after EGF induction. For A375 cells, we followed the criteria of 40% in gene expression after ints11 knockdown (see Supplemental Figs. 1, 6, 9; Supplemental Tables 1, 5, 6, 7). Heat maps were generated using SpotFire with Decision Site for Functional Genomics (SpotFire, Inc.).

### Gene set enrichment analysis (GSEA)

Gene expression in fold changes was obtained as described above, and the entire list of expressed genes was preranked and imported into the GSEA program (Subramanian et al. 2005) to perform GSEAs.

### Genome-wide identification of eRNA and SE RNA loci

For eRNA identification, we performed peak analysis from HeLa H3K27ac ChIP-seq data after EGF stimulation (GSE68401) using HOMER (Heinz et al. 2010) run in “histone” mode. ChromRNA-seq from HeLa cells (vehicle and EGF) was used for transcriptome assembly with Cufflinks version 2.2.1 (Trapnell et al. 2010) with the following options: -N -u --library-type fr-firststrand -g (RefSeq GTF file provided as guide) -M (rRNA, tRNA, and 7SK RNA mask file provided). We generated transcriptome assemblies for each of these samples separately and then used Cuffmerge to combine all annotations. We then removed all spliced transcripts and any transcript that overlapped or were in a window of ±2 kb of known RefSeq genes. Next, we used BEDTools (Quinlan and Hall 2010) to retain all pairs of transcripts that were head to head in a window of 500 nucleotides. We further selected the pair of transcripts whose TSS overlapped (±500 bp) with H3K27ac peaks. This eRNA annotation was merged with the RefSeq and used for all subsequent RNA-seq expression analyses. Seventy-five EGF-induced eRNAs located within 300 kb from the nearest EGF-responsive protein-coding genes were selected for analysis. For SEs, we used wild-type uninduced RNA-seq as “input” data and wild-type EGF-induced RNA-seq as “ChIP-seq” data. In total, 3051 peaks were detected, and, among them, 85 were called as SEs. After manually removing protein-coding regions from the 85 SEs, we had 36 bona fide SEs left. We combined these 36 SEs and 464 traditional enhancers to get the top 500 EGF-induced enhancers. They were ranked by their SE score: normalized peak score based on the highest peak score and the total number of peaks (3051). Next, we quantified tag counts at those 500 nonredundant peaks from RNA-seq data of shINTS11-treated, ERK inhibitor-treated, and INTS11 inhibitor-treated cells before and after EGF induction. As in the previous step, we normalized the EGF-induced tag to generate the SE score in the same way as wild-type samples.

### ChIP-seq data analysis and RNAPII traveling ratio

ChIP-seq data analysis was performed as described previously (Lai et al. 2015). Briefly, FastQ data were processed with Trimmomatic (Bolger et al. 2014) to remove low-quality reads and then were aligned to the human genome hg19 using Bowtie 2 (Langmead and Salzberg 2012). The bigWiggle file was generated with SAMtools and RseQC and then uploaded to the University of California at Santa Cruz Genome Browser. The average profile was generated with NGS Plot (Shen et al. 2014). RNAPII traveling ratio calculations were generated as described (Rahl et al. 2010). Briefly, RNAPII ChIP-seq density at the TSS (−30 bp to +300 bp) was divided by the read density over the rest of the gene body plus an additional 1 kb beyond the transcription end site (TES). The log_10_ (ratio) of genes (EGF, control, and ERK inhibitor treatment) was calculated using all different isoforms available in the Hg19 RefSeq annotation table that were considered express (FPKM > 1 in EGF treatment conditions) in our analysis.

### Antibodies

Antibodies used for CHIP and immunoblot included INTS11 (Bethyl Laboratories, A301-274A), INTS11 (Sigma, HPA029025), RNAPII (Santa Cruz Biotechnology, sc-899), GAPDH (Santa Cruz Biotechnology, sc-25778), phospo-ERK1/2 (Cell Signaling Technology, nos. 4370 and 9101; and Invitrogen, catalog no. 700012), ERK1/2 (Cell Signaling Technology, no. 9102), MED1 (Bethyl Laboratories, A300-793A), MED12 (Bethyl Laboratories, A300-774A), phospho-p90RSK (Thr359) (Cell Signaling Technology, no. 8753), RSK1 (Cell Signaling Technology, no. 9333), phosphor-EGFR (pTyr1068) (Cell Signaling Technology, no. 3777), EGF receptor (Cell Signaling Technology, no. 4267), and phosphothreonine (Cell Signaling Technology, no. 9381). Flag M2-conjugated beads (Sigma, A2220) were used for immunoprecipitation.

### Cell lines

Melanoma cell line A375 and lung cancer cell line A549 were purchased from American Type Culture Collection and maintained under suggested conditions. The RAF inhibitor-resistant line (A375R) was derived from A375 by culturing the cells in the medium containing RAF inhibitor (1 µM vemurafenib) for at least 3 mo.

### siRNA and plasmid transfections

Gene silencing was achieved by transfection of siRNAs (20 nM final concentration) in Optimem medium (Invitrogen) using Lipofectamine RNAiMax (Invitrogen, catalog no. 13778-100) according to the manufacturer's protocol. The siRNAs were purchased from Ambion (siINTS11#1 [catalog no. 29894], siINTS11#2 [catalog no. 29895], and negative control siRNA [catalog no. AM4611]) and Qiagen (negative control siRNA [catalog no. 1022076]). Plasmid expression mutant ERK2 or wild-type ERK2 and empty vector were transfected into the cells using Lipofectamine 3000 (Invitrogen, catalog no. L3000015) according to the manufacturer's protocol. The plasmid pBABEpuro-HA-ERK2-Mut was a gift from Christopher Counter (Addgene, plasmid no. 53203) (Brady et al. 2014).

### Compounds

Vemurafenib (S1267), PD0325901 (S1036), SCH772984 (S7101), and LY294002 (S1105), purchased from Selleck Chemicals, were resuspended in DMSO.

### RNA extraction and ChIP

The cells were maintained in 0.5% FBS-containing medium for 48 h and then subjected to EGF stimulation for 20 min by adding 100 ng/mL EGF (Thermo Fisher Scientific, PHG0311L). For inhibitor treatment, the cells were incubated in medium containing 1 µM vemurafenib, 200 nM PD0325901, or 1 µM SCH772984 for 3 h before collection. The chromatin-associated RNA fraction was prepared as described previously (Lai et al. 2015). To prepare the samples for ChIP, the cells were fixed with 1% formaldehyde for 10 min in a culture dish followed by incubation in 0.125 M glycine at room temperature to halt the fixation. The cells were washed, scraped, and pelleted in cold PBS. ChIP was performed as described previously (Lai et al. 2015).

### Cell growth curve and RAF inhibitor IC50 (concentration of 50% growth inhibition)

Exponentially growing cells were trypsinized and counted with a Moxi Z miniautomated cell counter (ORFLO Technologies). The cells were seeded into a 12-well plate at a density of 2 × 10^4^ to ∼4 × 10^4^ cells per well and then treated after overnight incubation. Lung cancer cell line A549 and melanoma cell line A375 were transfected with nontargeting siRNA or siRNA against INTS11 (shINTS11 #1 + #2). HeLa cells harboring an inducible shRNA cassette were cultured in medium containing doxycycline to knock down INTS11. The cells stably expressing Flag-INTS11 refractory siRNAs were used to restore the INTS11 protein level. To test MAP kinase pathway inhibitors on cell growth inhibition, A549 and HeLa cells were treated with MEK inhibitor at 0.2 and 0.5 µM or ERK inhibitor at 0.5 and 1 µM, respectively. A375 and RAF inhibitor-resistant line A375R were treated with RAF inhibitor at 0.4 µM. The cells were trypsinized and counted at days of treatment as indicted. To measure the growth inhibition effect of vemurafenib (IC50), the cells were plated into a 96-well plate and then treated with vehicle or RAF inhibitor for 3 d. PrestoBlu cell viability reagent (ThermoFisher Scientific, catalog no. A13261) was used to measure cell viability according to the manufacturer's protocol. GraphPad Prism software was used to generate graphs and calculate IC50s.

### Primers for ChIP-qPCR (ChIP combined with qPCR)

Primers for ChIP-qPCR were as follows: NR4A1-TSS forward (5′-GAGCGCTTAAGAGGAGGGTC-3′), NR4A1-TSS reverse (5′-GCACTCCCCCAAGTTTCGTA-3′), NR4A1-TSS forward2 (5′-ACGGAGCGCTTAAGAGGAG-3′), NR4A1-TSS reverse2 (5′-CTCCCGAAGTTCTTCTGTGC-3′), EGR1-TSS forward (5′-GTCCTGCCATATTAGGGCTTCC-3′), EGR1-TSS reverse: 5′-TATTTGAAGGGTCTGGAACGGC-3′), EGR1-TSS forward2 (5′-TGCAGATCTCTGACCCGTTC-3′), EGR1-TSS reverse2 (5′-TCATCTCCTCCAGCTTAGGG-3′), EGR1downstream (∼3 kb from TES) forward (5′-AAAACCAAGGGCACGAGACA-3′), EGR1 downstream (∼3 kb from TES) reverse (5′-GTTCAACACTCTCCGGGACC-3′), FOSB-TSS forward (5′-ATAAATACAGGCTGGCGGGT-3′), FOSB-TSS reverse (5′-AAGTCTTGGTTCCGCGTGTC-3′), EHD1-TSS forward (5′-CCCCATTGGCTGATTCCAAAT-3′), and EHD1-TSS reverse (5′-CTTCCTAACCGCAGCACTTTC-3′).

### Accession numbers

All of the genome-wide data of this study have been deposited in the NCBI Gene Expression Omnibus (GEO) database (GSE85089).

## Supplementary Material

Supplemental Material

## References

[YUEGAD301697C1] Bolger AM, Lohse M, Usadel B. 2014 Trimmomatic: a flexible trimmer for Illumina sequence data. Bioinformatics 30: 2114–2120. 24695404 10.1093/bioinformatics/btu170 PMC4103590

[YUEGAD301697C2] Brady DC, Crowe MS, Turski ML, Hobbs GA, Yao X, Chaikuad A, Knapp S, Xiao K, Campbell SL, Thiele DJ, 2014 Copper is required for oncogenic BRAF signalling and tumorigenesis. Nature 509: 492–496. 24717435 10.1038/nature13180 PMC4138975

[YUEGAD301697C3] Bryant KL, Mancias JD, Kimmelman AC, Der CJ. 2014 KRAS: feeding pancreatic cancer proliferation. Trends Biochem Sci 39: 91–100. 24388967 10.1016/j.tibs.2013.12.004 PMC3955735

[YUEGAD301697C4] Chen RH, Sarnecki C, Blenis J. 1992 Nuclear localization and regulation of erk- and rsk-encoded protein kinases. Mol Cell Biol 12: 915–927. 1545823 10.1128/mcb.12.3.915-927.1992 PMC369523

[YUEGAD301697C5] Davies H, Bignell GR, Cox C, Stephens P, Edkins S, Clegg S, Teague J, Woffendin H, Garnett MJ, Bottomley W, 2002 Mutations of the BRAF gene in human cancer. Nature 417: 949–954. 12068308 10.1038/nature00766

[YUEGAD301697C6] Davis S, Vanhoutte P, Pages C, Caboche J, Laroche S. 2000 The MAPK/ERK cascade targets both Elk-1 and cAMP response element-binding protein to control long-term potentiation-dependent gene expression in the dentate gyrus in vivo. J Neurosci 20: 4563–4572. 10844026 10.1523/JNEUROSCI.20-12-04563.2000 PMC6772466

[YUEGAD301697C7] Dhillon AS, Hagan S, Rath O, Kolch W. 2007 MAP kinase signalling pathways in cancer. Oncogene 26: 3279–3290. 17496922 10.1038/sj.onc.1210421

[YUEGAD301697C8] Donner AJ, Ebmeier CC, Taatjes DJ, Espinosa JM. 2010 CDK8 is a positive regulator of transcriptional elongation within the serum response network. Nat Struct Mol Biol 17: 194–201. 20098423 10.1038/nsmb.1752 PMC2920286

[YUEGAD301697C9] Foulds CE, Nelson ML, Blaszczak AG, Graves BJ. 2004 Ras/mitogen-activated protein kinase signaling activates Ets-1 and Ets-2 by CBP/p300 recruitment. Mol Cell Biol 24: 10954–10964. 15572696 10.1128/MCB.24.24.10954-10964.2004 PMC533975

[YUEGAD301697C10] Galbraith MD, Saxton J, Li L, Shelton SJ, Zhang H, Espinosa JM, Shaw PE. 2013 ERK phosphorylation of MED14 in promoter complexes during mitogen-induced gene activation by Elk-1. Nucleic Acids Res 41: 10241–10253. 24049075 10.1093/nar/gkt837 PMC3905876

[YUEGAD301697C11] Gardini A, Baillat D, Cesaroni M, Hu D, Marinis JM, Wagner EJ, Lazar MA, Shilatifard A, Shiekhattar R. 2014 Integrator regulates transcriptional initiation and pause release following activation. Mol Cell 56: 128–139. 25201415 10.1016/j.molcel.2014.08.004 PMC4292851

[YUEGAD301697C12] Garnett MJ, Marais R. 2004 Guilty as charged: B-RAF is a human oncogene. Cancer Cell 6: 313–319. 15488754 10.1016/j.ccr.2004.09.022

[YUEGAD301697C13] Gonzalez FA, Seth A, Raden DL, Bowman DS, Fay FS, Davis RJ. 1993 Serum-induced translocation of mitogen-activated protein kinase to the cell surface ruffling membrane and the nucleus. J Cell Biol 122: 1089–1101. 8394846 10.1083/jcb.122.5.1089 PMC2119622

[YUEGAD301697C14] Heinz S, Benner C, Spann N, Bertolino E, Lin YC, Laslo P, Cheng JX, Murre C, Singh H, Glass CK. 2010 Simple combinations of lineage-determining transcription factors prime *cis*-regulatory elements required for macrophage and B cell identities. Mol Cell 38: 576–589. 20513432 10.1016/j.molcel.2010.05.004 PMC2898526

[YUEGAD301697C15] Janknecht R, Nordheim A. 1996 MAP kinase-dependent transcriptional coactivation by Elk-1 and its cofactor CBP. Biochem Biophys Res Commun 228: 831–837. 8941362 10.1006/bbrc.1996.1740

[YUEGAD301697C16] Jun JH, Yoon WJ, Seo SB, Woo KM, Kim GS, Ryoo HM, Baek JH. 2010 BMP2-activated Erk/MAP kinase stabilizes Runx2 by increasing p300 levels and histone acetyltransferase activity. J Biol Chem 285: 36410–36419. 20851880 10.1074/jbc.M110.142307 PMC2978570

[YUEGAD301697C17] Karin M, Hunter T. 1995 Transcriptional control by protein phosphorylation: signal transmission from the cell surface to the nucleus. Curr Biol 5: 747–757. 7583121 10.1016/s0960-9822(95)00151-5

[YUEGAD301697C18] Kim D, Pertea G, Trapnell C, Pimentel H, Kelley R, Salzberg SL. 2013 TopHat2: accurate alignment of transcriptomes in the presence of insertions, deletions and gene fusions. Genome Biol 14: R36. 23618408 10.1186/gb-2013-14-4-r36 PMC4053844

[YUEGAD301697C19] Lai F, Gardini A, Zhang A, Shiekhattar R. 2015 Integrator mediates the biogenesis of enhancer RNAs. Nature 525: 399–403. 26308897 10.1038/nature14906 PMC4718573

[YUEGAD301697C20] Langmead B, Salzberg SL. 2012 Fast gapped-read alignment with Bowtie 2. Nat Methods 9: 357–359. 22388286 10.1038/nmeth.1923 PMC3322381

[YUEGAD301697C21] Murphy LO, Smith S, Chen RH, Fingar DC, Blenis J. 2002 Molecular interpretation of ERK signal duration by immediate early gene products. Nat Cell Biol 4: 556–564. 12134156 10.1038/ncb822

[YUEGAD301697C22] Nelson ML, Kang HS, Lee GM, Blaszczak AG, Lau DK, McIntosh LP, Graves BJ. 2010 Ras signaling requires dynamic properties of Ets1 for phosphorylation-enhanced binding to coactivator CBP. Proc Natl Acad Sci 107: 10026–10031. 20534573 10.1073/pnas.0915137107 PMC2890480

[YUEGAD301697C23] Pandey PK, Udayakumar TS, Lin X, Sharma D, Shapiro PS, Fondell JD. 2005 Activation of TRAP/mediator subunit TRAP220/Med1 is regulated by mitogen-activated protein kinase-dependent phosphorylation. Mol Cell Biol 25: 10695–10710. 16314496 10.1128/MCB.25.24.10695-10710.2005 PMC1316958

[YUEGAD301697C24] Quinlan AR, Hall IM. 2010 BEDTools: a flexible suite of utilities for comparing genomic features. Bioinformatics 26: 841–842. 20110278 10.1093/bioinformatics/btq033 PMC2832824

[YUEGAD301697C25] Rahl PB, Lin CY, Seila AC, Flynn RA, McCuine S, Burge CB, Sharp PA, Young RA. 2010 c-Myc regulates transcriptional pause release. Cell 141: 432–445. 20434984 10.1016/j.cell.2010.03.030 PMC2864022

[YUEGAD301697C26] Roberts PJ, Der CJ. 2007 Targeting the Raf–MEK–ERK mitogen-activated protein kinase cascade for the treatment of cancer. Oncogene 26: 3291–3310. 17496923 10.1038/sj.onc.1210422

[YUEGAD301697C27] Roux PP, Blenis J. 2004 ERK and p38 MAPK-activated protein kinases: a family of protein kinases with diverse biological functions. Microbiol Mol Biol Rev 68: 320–344. 15187187 10.1128/MMBR.68.2.320-344.2004 PMC419926

[YUEGAD301697C28] Rowan BG, Weigel NL, O'Malley BW. 2000 Phosphorylation of steroid receptor coactivator-1. Identification of the phosphorylation sites and phosphorylation through the mitogen-activated protein kinase pathway. J Biol Chem 275: 4475–4483. 10660621 10.1074/jbc.275.6.4475

[YUEGAD301697C29] Samatar AA, Poulikakos PI. 2014 Targeting RAS–ERK signalling in cancer: promises and challenges. Nat Rev Drug Discov 13: 928–942. 25435214 10.1038/nrd4281

[YUEGAD301697C30] Santarpia L, Lippman SM, El-Naggar AK. 2012 Targeting the MAPK–RAS–RAF signaling pathway in cancer therapy. Expert Opin Ther Targets 16: 103–119. 22239440 10.1517/14728222.2011.645805 PMC3457779

[YUEGAD301697C31] Shen L, Shao N, Liu X, Nestler E. 2014 ngs.plot: quick mining and visualization of next-generation sequencing data by integrating genomic databases. BMC Genomics 15: 284. 24735413 10.1186/1471-2164-15-284 PMC4028082

[YUEGAD301697C32] Subramanian A, Tamayo P, Mootha VK, Mukherjee S, Ebert BL, Gillette MA, Paulovich A, Pomeroy SL, Golub TR, Lander ES, 2005 Gene set enrichment analysis: a knowledge-based approach for interpreting genome-wide expression profiles. Proc Natl Acad Sci 102: 15545–15550. 16199517 10.1073/pnas.0506580102 PMC1239896

[YUEGAD301697C33] Tee WW, Shen SS, Oksuz O, Narendra V, Reinberg D. 2014 Erk1/2 activity promotes chromatin features and RNAPII phosphorylation at developmental promoters in mouse ESCs. Cell 156: 678–690. 24529373 10.1016/j.cell.2014.01.009 PMC4006806

[YUEGAD301697C34] Trapnell C, Williams BA, Pertea G, Mortazavi A, Kwan G, van Baren MJ, Salzberg SL, Wold BJ, Pachter L. 2010 Transcript assembly and quantification by RNA-seq reveals unannotated transcripts and isoform switching during cell differentiation. Nat Biotechnol 28: 511–515. 20436464 10.1038/nbt.1621 PMC3146043

[YUEGAD301697C35] Wang G, Balamotis MA, Stevens JL, Yamaguchi Y, Handa H, Berk AJ. 2005 Mediator requirement for both recruitment and postrecruitment steps in transcription initiation. Mol Cell 17: 683–694. 15749018 10.1016/j.molcel.2005.02.010

[YUEGAD301697C36] Yoon S, Seger R. 2006 The extracellular signal-regulated kinase: multiple substrates regulate diverse cellular functions. Growth Factors 24: 21–44. 16393692 10.1080/02699050500284218

