# Supplementary material for: Integrator orchestrates RAS/ERK1/2 signaling transcriptional programs
Source: Genes Dev. 2017 Sep 1;31(17):1809–20. doi: 10.1101/gad.301697.117 (PMC5666678; doi:10.1101/gad.301697.117)
Supplement: Supplemental Material [file supp_31_17_1809__index.html]

Supplemental Material 

# Integrator orchestrates RAS/ERK1/2 signaling transcriptional programs

## Supplemental Material

- Supplemental\_Fig\_S7.pdf
- Supplemental\_Fig\_S5.pdf
- Supplemental\_Fig\_S9.pdf
- Supplemental\_Fig\_S3.pdf
- Supplemental\_Fig\_S1.pdf
- Supplemental\_Fig\_S8.pdf
- Supplemental\_Fig\_S6.pdf
- Supplemental\_Fig\_S10.pdf
- Supplemental\_Fig\_S4.pdf
- Supplemental\_Fig\_S2.pdf
- Supplemental\_Table\_6\_a549\_EGF\_Responsive\_genes.xlsx
- Supplemental\_Table\_7\_a375\_MAPK\_Inhibitors\_genes.xlsx
- Supplemental\_Legends.docx
- Supplemental\_Table\_2\_HeLa\_eRNAs.xlsx
- Supplemental\_Table\_1\_HeLa\_EGF\_responsive\_genes.xlsx
- Supplemental\_Table\_4\_FPKM\_values\_HeLa\_EGF\_genes.xlsx
- Supplemental\_Table\_3\_HeLa\_SuperEnhancers.xlsx
- Supplemental\_Table\_5\_Hela\_serum.xlsx
