# Supplementary figures and images for: Integrator orchestrates RAS/ERK1/2 signaling transcriptional programs
Source: Genes Dev. 2017 Sep 1;31(17):1809–20. doi: 10.1101/gad.301697.117 (PMC5666678; doi:10.1101/gad.301697.117)

**A****RNAP II**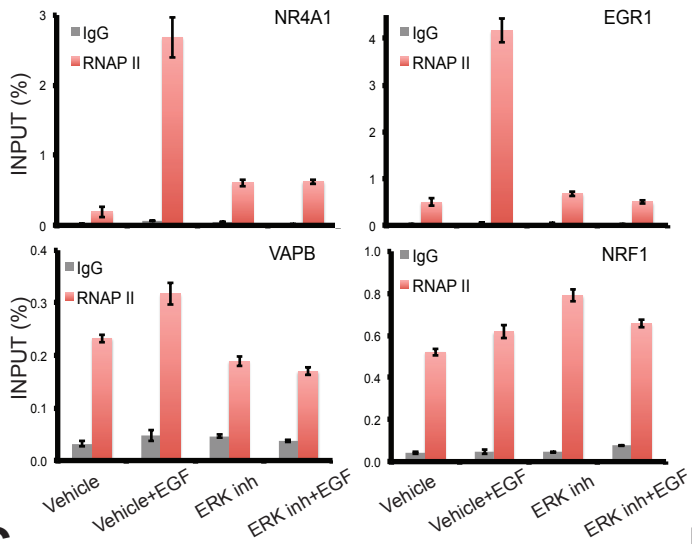**B****INTS11**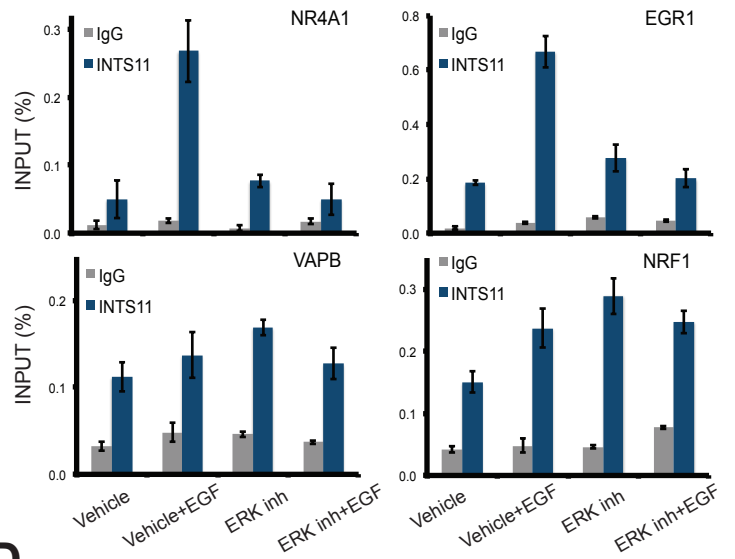**C****MED1**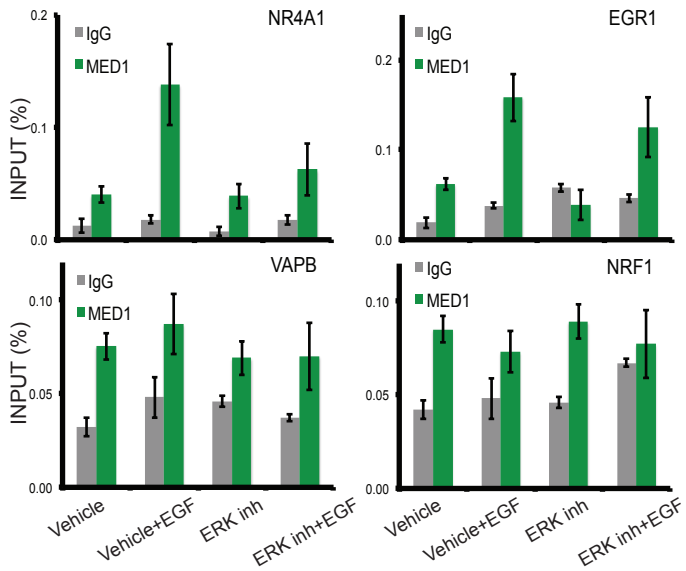**D****MED12**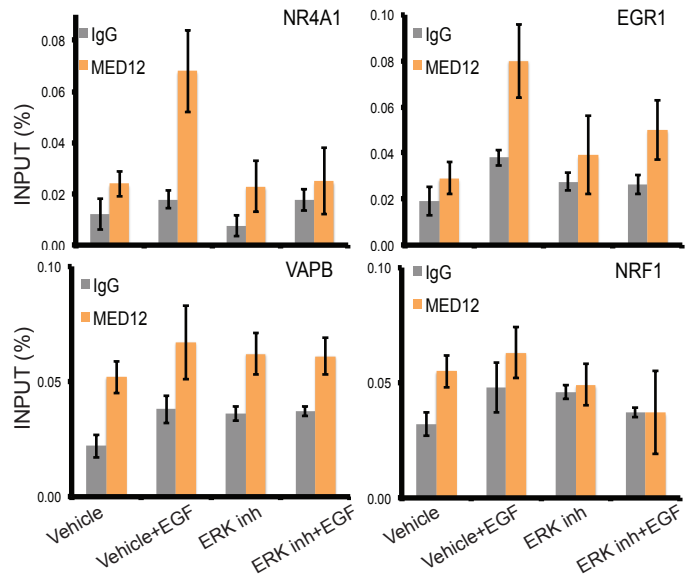

Supplement: Supplemental Material [file supp_31.17.1809_Supplemental_Fig_S7.pdf]

**A**

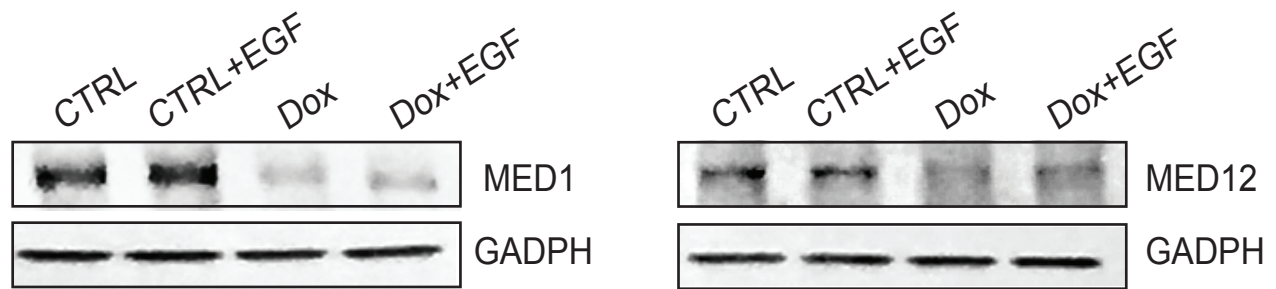

**B**

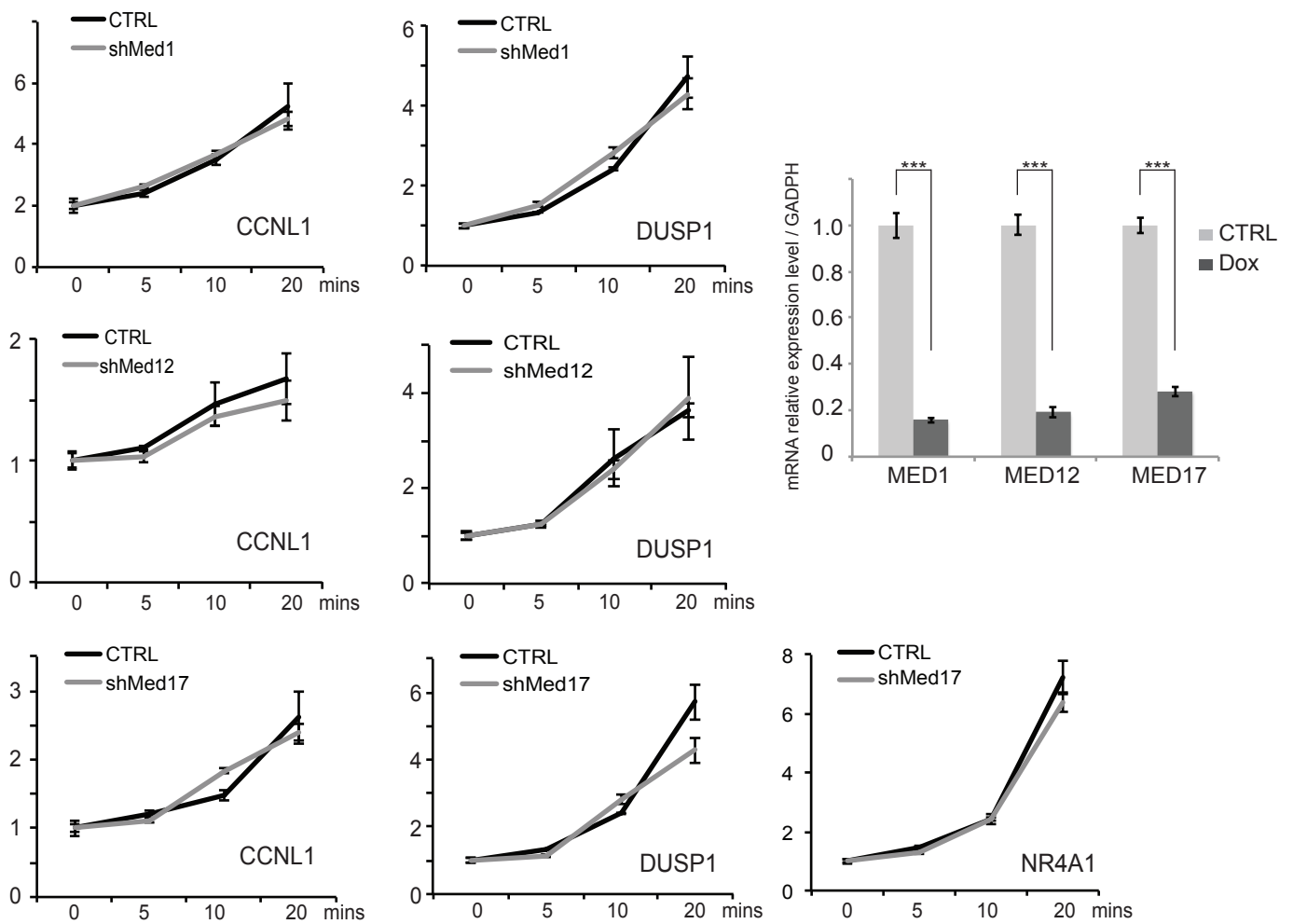

Supplement: Supplemental Material [file supp_31.17.1809_Supplemental_Fig_S5.pdf]

A

HeLa

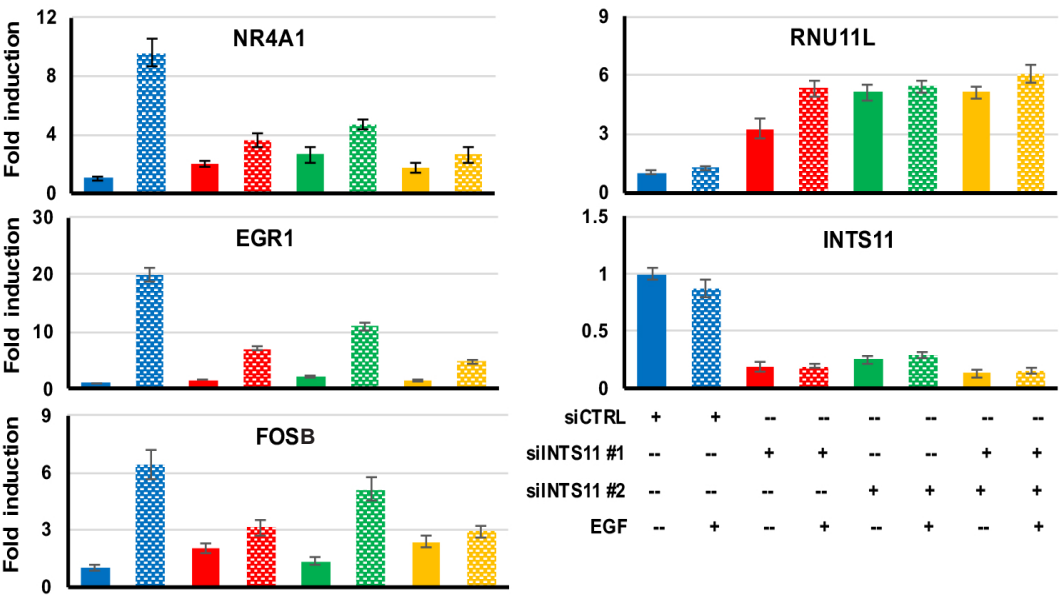

B

A549

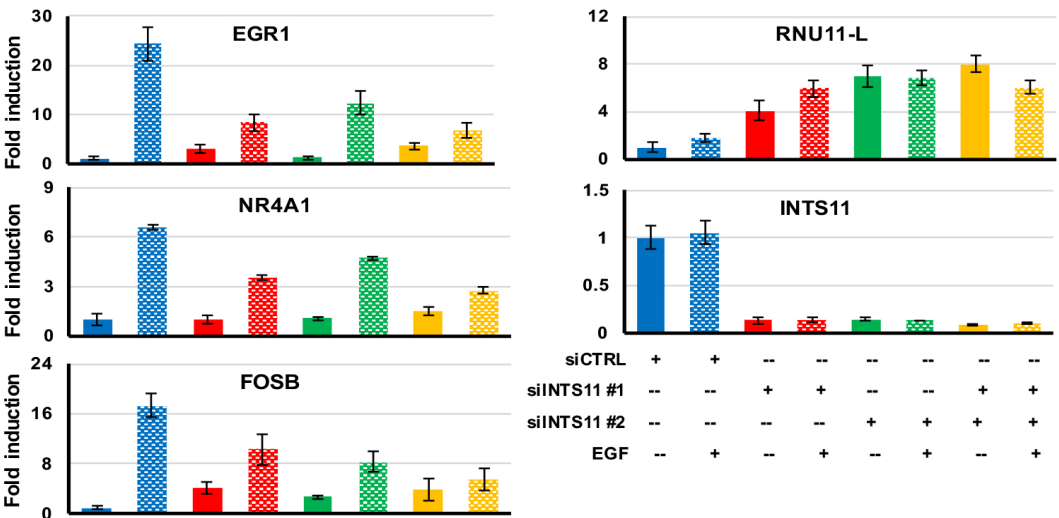

C

A375

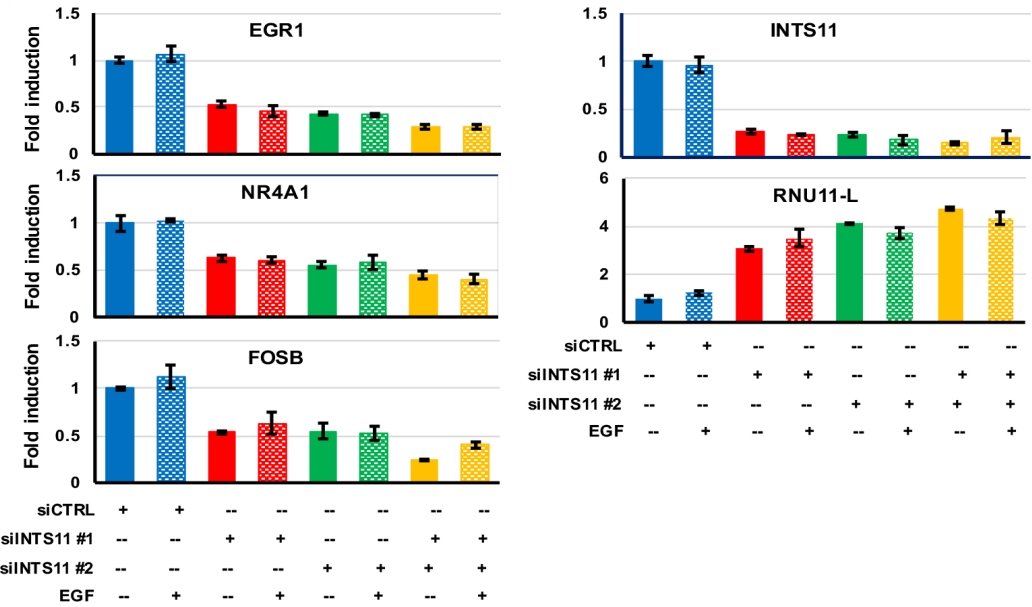

Supplement: Supplemental Material [file supp_31.17.1809_Supplemental_Fig_S3.pdf]

**A**

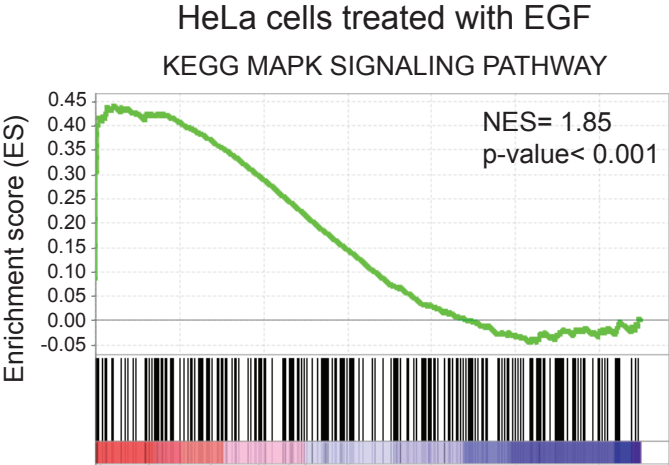

**B**

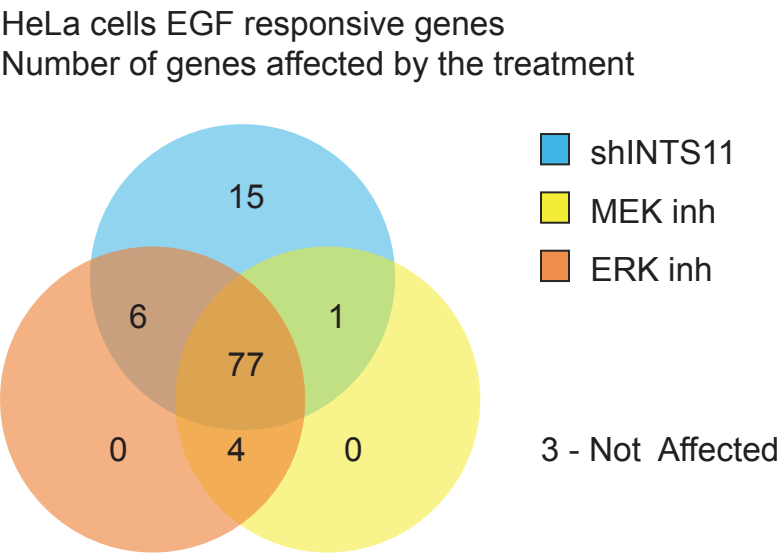

Supplement: Supplemental Material [file supp_31.17.1809_Supplemental_Fig_S1.pdf]

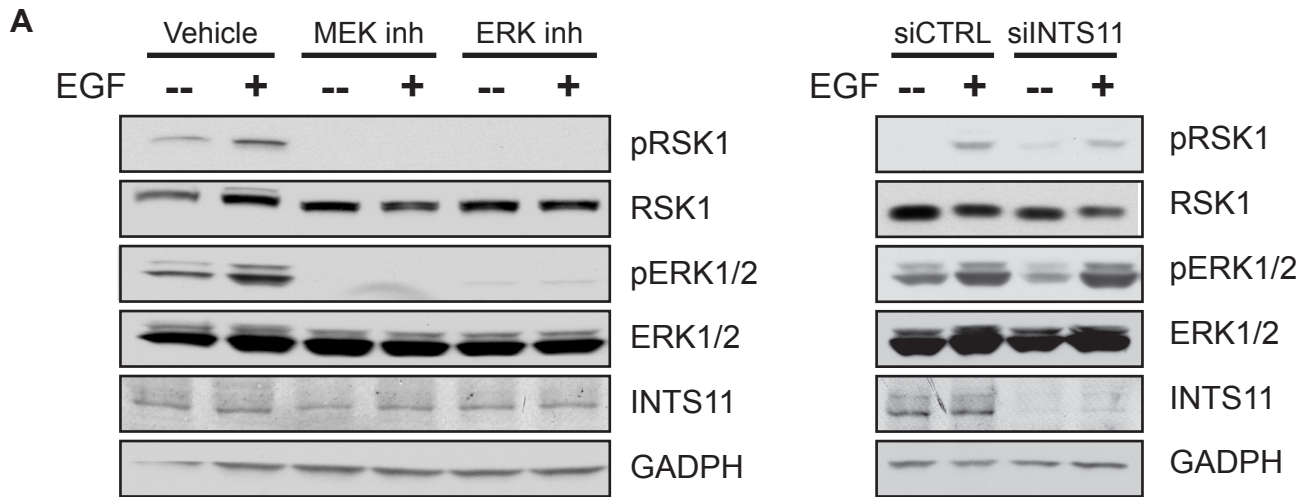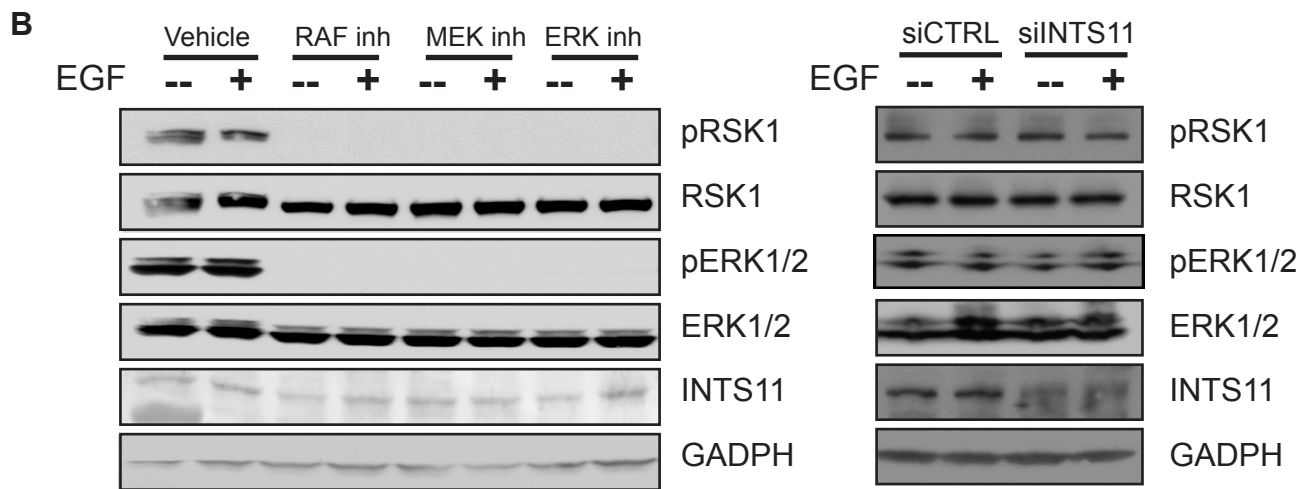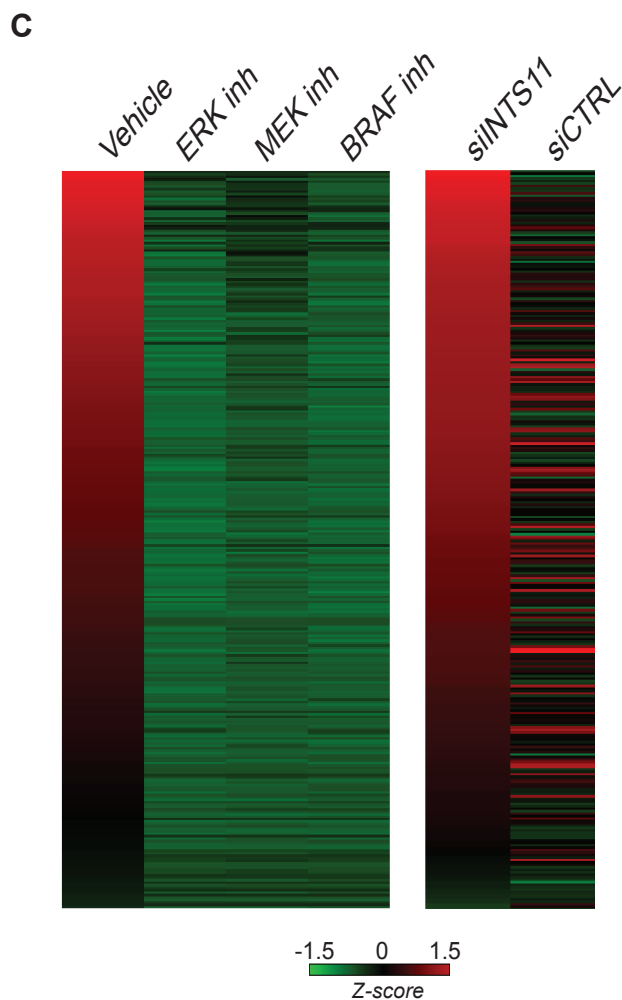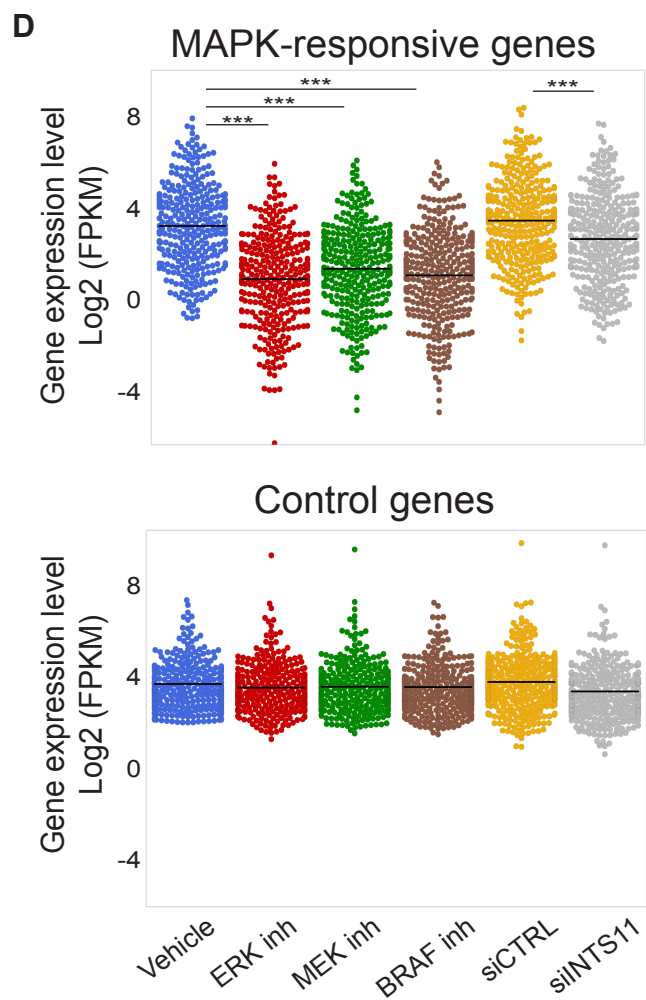

Supplement: Supplemental Material [file supp_31.17.1809_Supplemental_Fig_S8.pdf]

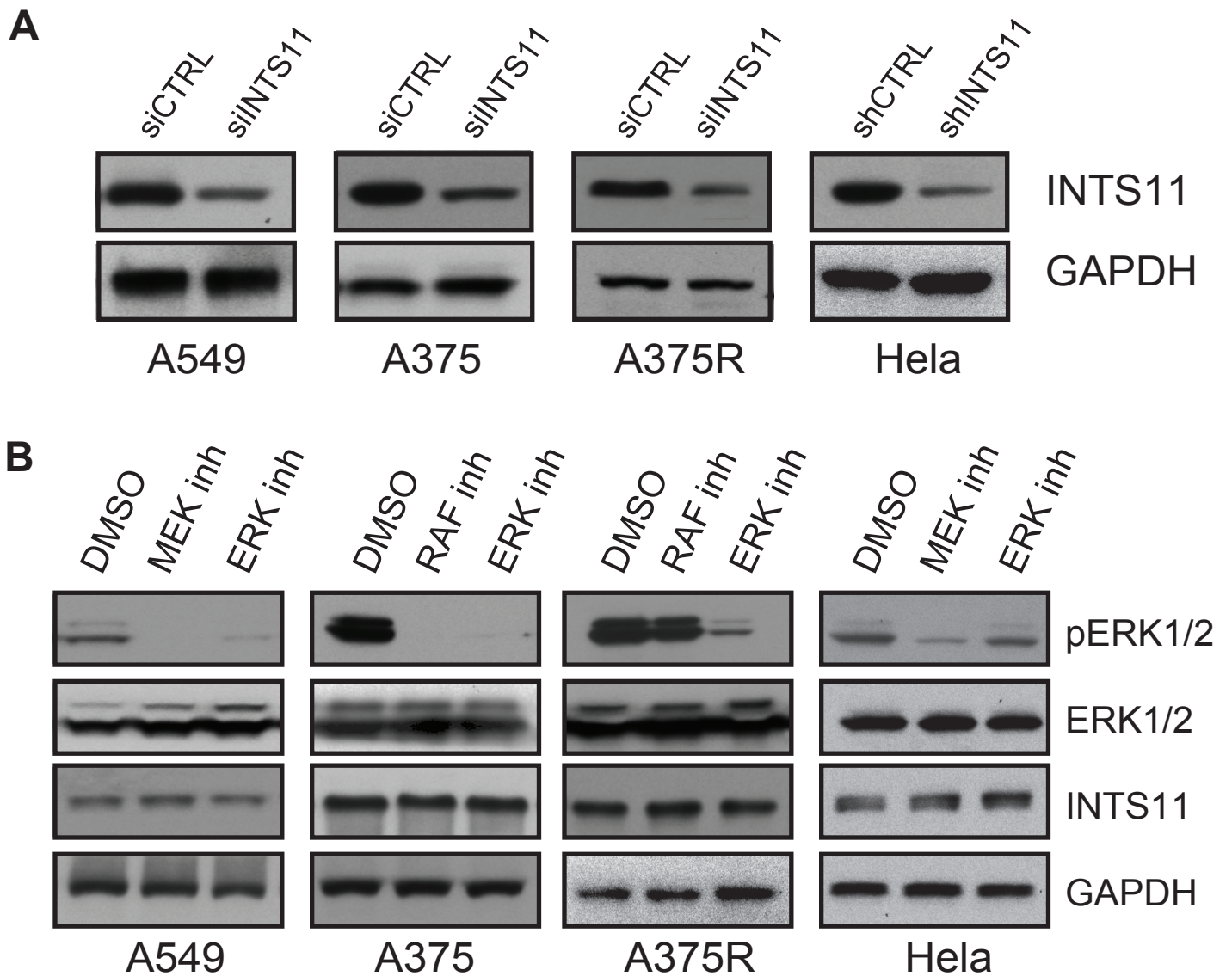

Supplement: Supplemental Material [file supp_31.17.1809_Supplemental_Fig_S10.pdf]

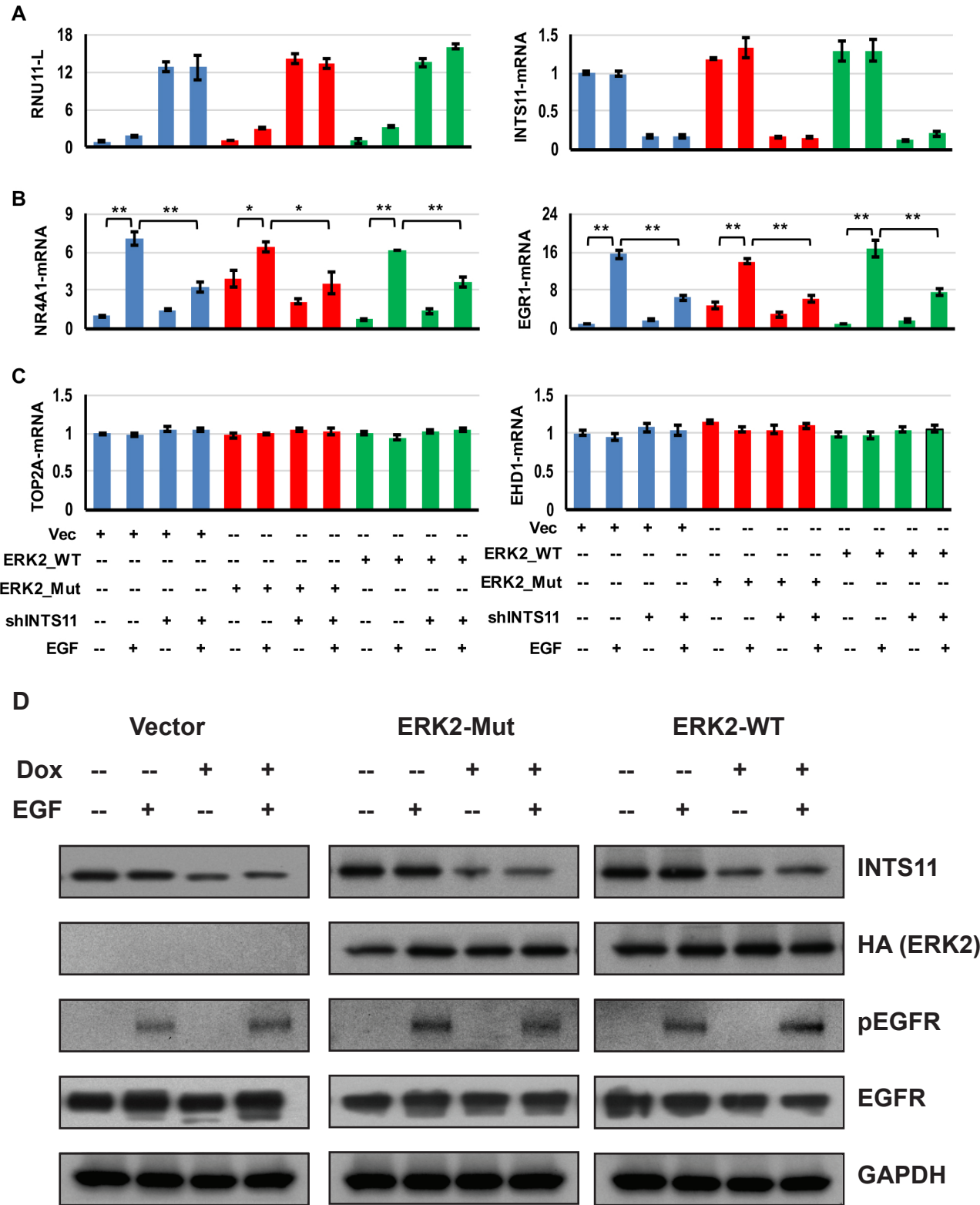

Supplement: Supplemental Material [file supp_31.17.1809_Supplemental_Fig_S4.pdf]

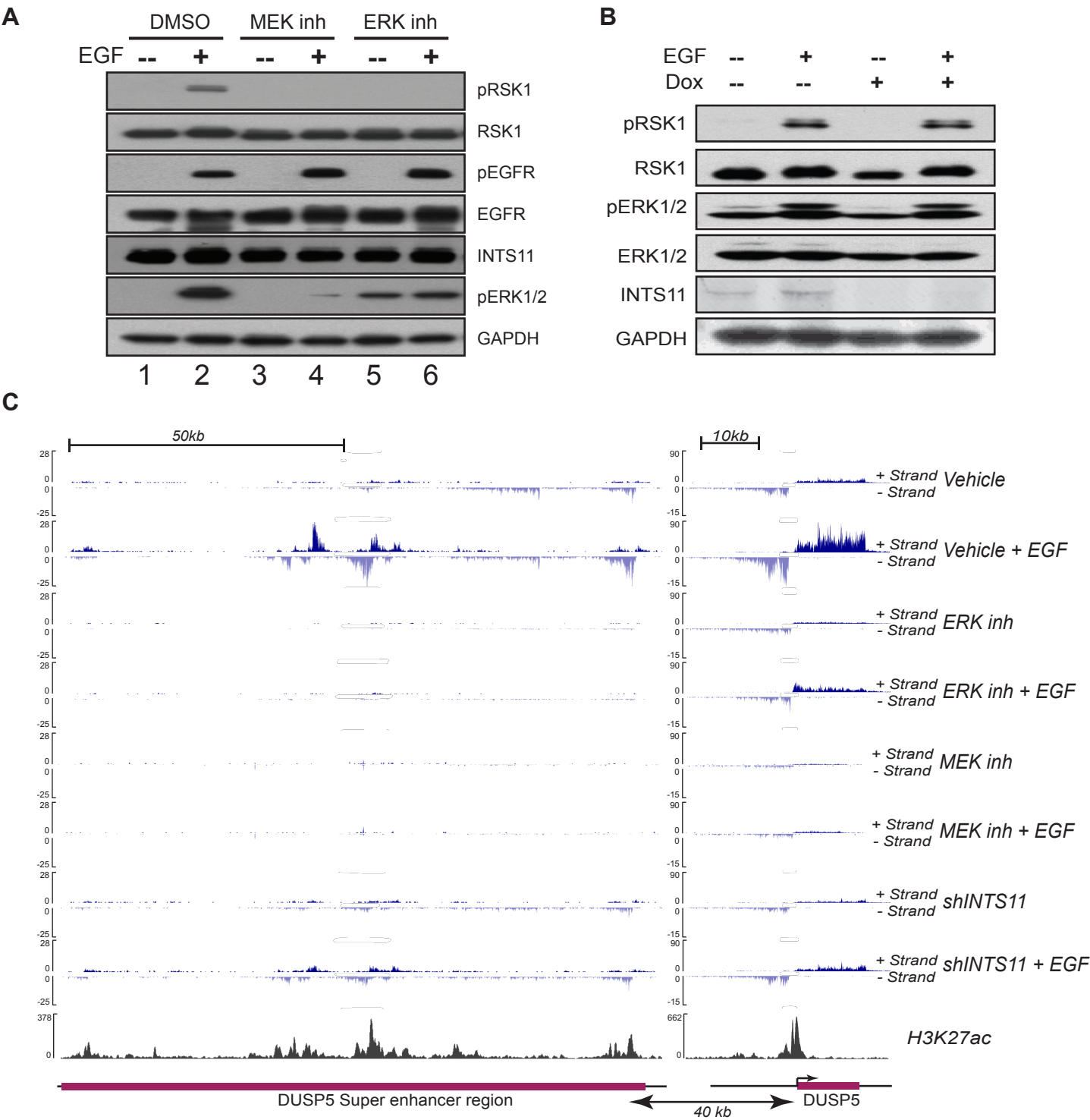

Supplement: Supplemental Material [file supp_31.17.1809_Supplemental_Fig_S2.pdf]
