# Supplementary material for: Integrator orchestrates RAS/ERK1/2 signaling transcriptional programs
Source: Genes Dev. 2017 Sep 1;31(17):1809–20. doi: 10.1101/gad.301697.117 (PMC5666678; doi:10.1101/gad.301697.117)
Supplement: Supplemental Material [file supp_31.17.1809_Supplemental_Fig_S9.pdf]

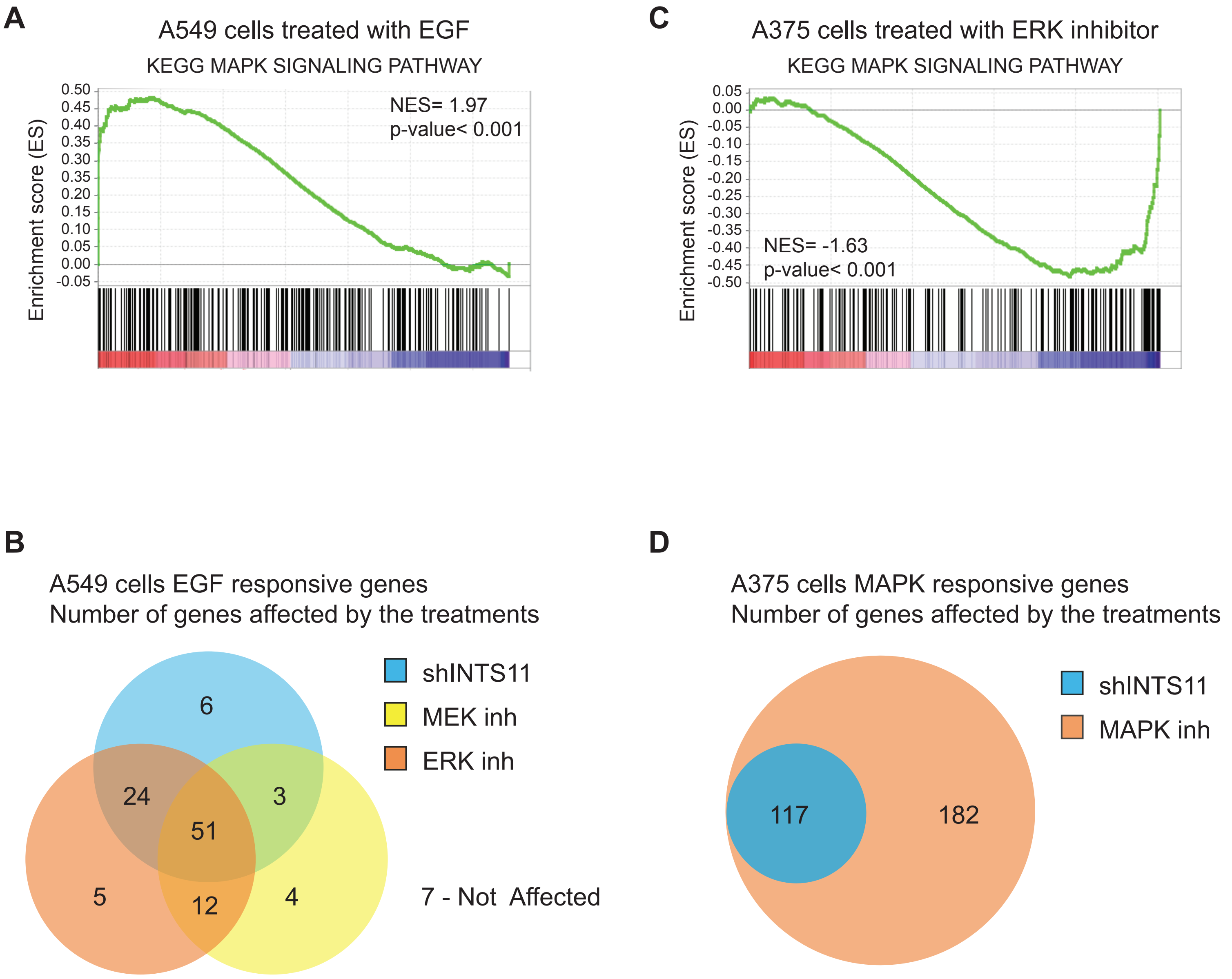
A
A549 cells treated with EGF
KEGG MAPK SIGNALING PATHWAY
NES= 1.97
p-value< 0.001
Enrichment score (ES)
0.50
0.45
0.40
0.35
0.30
0.35
0.20
0.15
0.10
0.05
0.00
-0.05
C
A375 cells treated with ERK inhibitor
KEGG MAPK SIGNALING PATHWAY
NES= -1.63
p-value< 0.001
Enrichment score (ES)
0.05
0.00
-0.05
-0.10
-0.15
-0.20
-0.25
-0.30
-0.35
-0.40
-0.45
-0.50
B
A549 cells EGF responsive genes
Number of genes affected by the treatments
shINTS11
MEK inh
ERK inh
6
24
3
51
5
12
4
7 - Not Affected
D
A375 cells MAPK responsive genes
Number of genes affected by the treatments
shINTS11
MAPK inh
117
182
