# Supplementary material for: Integrator orchestrates RAS/ERK1/2 signaling transcriptional programs
Source: Genes Dev. 2017 Sep 1;31(17):1809–20. doi: 10.1101/gad.301697.117 (PMC5666678; doi:10.1101/gad.301697.117)

Jingyin_Supplemental_Fig_S6

A

HeLa cells EGF responsive genes
Number of genes affected by the treatment

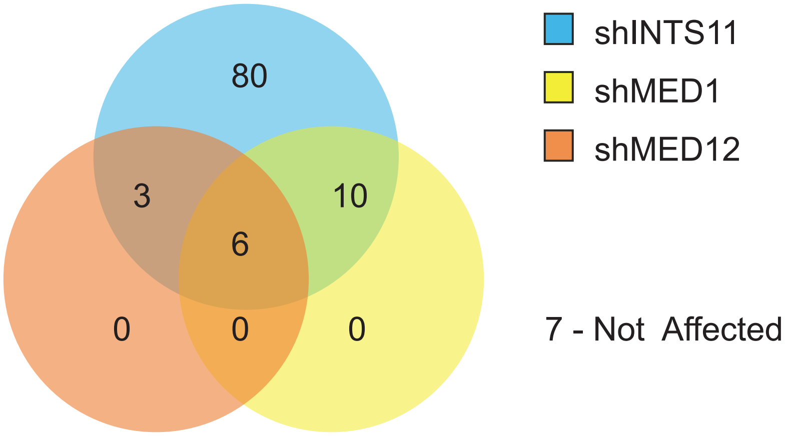

B

HeLa cells EGF responsive genes
Number of genes affected by the treatment

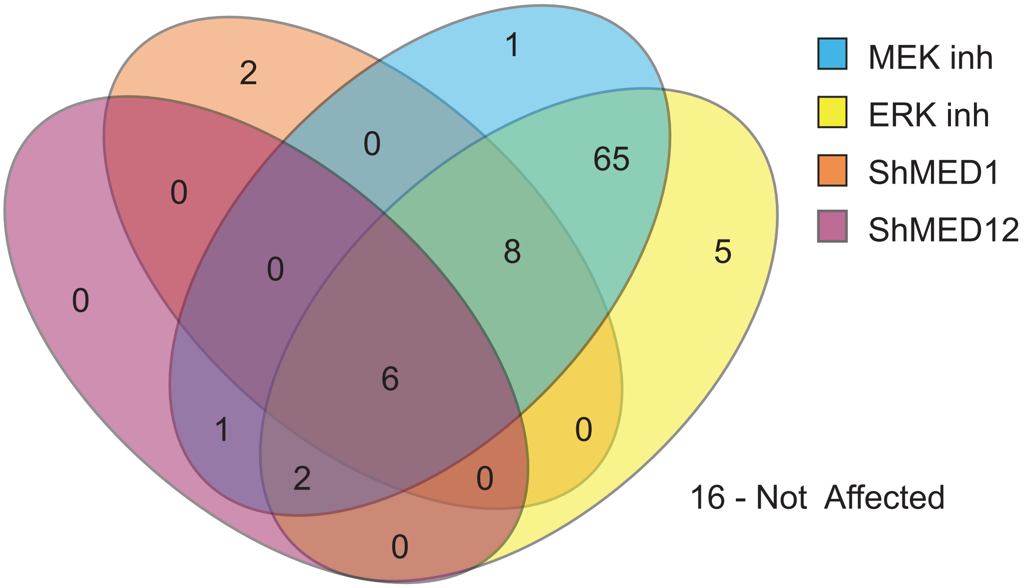

Supplement: Supplemental Material [file supp_31.17.1809_Supplemental_Fig_S6.pdf]
