# Supplementary material for: Integrator orchestrates RAS/ERK1/2 signaling transcriptional programs
Source: Genes Dev. 2017 Sep 1;31(17):1809–20. doi: 10.1101/gad.301697.117 (PMC5666678; doi:10.1101/gad.301697.117)
Supplement: Supplemental Material [file supp_31.17.1809_Supplemental_Legends.docx]

**Supplemental Figures**

**Supplemental Fig 1. Integrator affects MAPK-mediated transcriptional response.** *(A)* GSEA analyses reveal a positive enrichment in MAPK signaling pathways in HeLa cells under EGF stimulus. *(B)* Venn diagram showing 106 EGF responsive genes in HeLa cells overlap with INTS11 KD, MEK inhibitor or ERK inhibitor after EGF stimulus.

**Supplemental Fig 2. ERK1/2 or MEK inhibition attenuates EGF transcriptional responsiveness in HeLa cells.** *(A)* Immunoblot of HeLa cells treated with Vehicle, MEK (PD0325901) or ERK inhibitor (SCH772984) for 3 hours. The cells were collected before and after 20 minutes of EGF induction. *(B)* Immunoblot of HeLa cells transduced with doxycycline-inducible shRNAs targeting INTS11 before and after 20 minutes of EGF induction. *(C)* ChromRNA-seq reveals ERK inhibition, MEK inhibition or INTS11 knockdown restrain transcriptional activation of Super-Enhancer and protein coding regions at *DUSP5 locus*.

**Supplemental Fig 3. Targeting INTS11 with RNAi specifically affect MAP kinase signaling.** *(A-C)* HeLa, KRAS mutant lung cancer cell A549 and BRAF mutant melanoma cell A375 were transfected with non-targeting siRNA (siCTRL) or single siRNA or double siRNAs (siINTS11 #1 and #2) against INTS11 as indicated in Figure. Chromatin associated RNA was extracted before or after 20 min of EGF stimulation. RT-qPCR was performed with specific primer pairs to detect mRNA levels of NR4A1, EGR1, FOSB, INTS11 genes and RNA transcript level of unprocessed RNU11 gene (RNU11L). Both INTS11 specific siRNAs effectively knock down INTS11 gene and cause usnRNA processing defect and further block MAP kinase signaling. Combination of two siRNAs has stronger phenotype than ether of single siRNA. Shown in Figure was the average of three independent experiments.

**Supplemental Fig 4. Targeting Integrator diminishes the activity of the gain-of-function mutant ERK2.** *(A-C)* RT-qPCR measuring the transcript levels of RNU11L, INTS11, NR4A1, EGR1, TOP2A and EHD1 genes. Shown in Figures was the average of 4 times of qPCR results from 2 independent experiments. (*) P<0.05 (**) P<0.01. *(D)* Western blot of the whole cell extract to check INTS11, HA-ERK2-Mut and HA-ERK2-WT expression level and the activation of EGFR (Cell signaling, pTyr1068 #3777) after the EGF stimulation. GAPDH was used as loading control. HeLa cells harboring inducible INTS11-shRNA were transfected with empty vector, plasmid expressing HA-ERK2-Mut or HA-ERK2-WT. The cells were undergoing Doxycycline treatment (1µg/ml) for three days to downregulate INTS11 and serum depleted for two days. The chromatin associated RNA and whole cell lysate were prepared before or after 20min EFG stimulation.

**Supplemental Fig 5. Depletion of Mediator subunits does not alter the EGF-induced gene transcriptional activation.** *(A)* Total cell lysates were immunoblotted with MED1 (left) or MED12 (right) specific antibodies in corresponding doxycycline-inducible shRNA clones. *(B)* Knockdown of MED1, MED12 or MED17 does not affect EGF-induced gene activation. The change of gene transcription was followed throughout a 20 min time-course after EGF induction by qRT-PCR. Error bars represent ± SEM (n=3 biological independent experiments). (***) *P*<0.01 by two-sided *t*-test.

**Supplemental Fig 6. Mediator does not significantly impact MAPK-mediated transcriptional response.** *(A)* Venn diagram showing 106 EGF responsive genes in HeLa cells overlap of INTS11 KD, shMED1 and shMED12 under EGF stimulus. *(B)* Venn diagram showing 106 EGF responsive genes in HeLa cells overlap of MEK inhibitor, ERK inhibitor, shMED1 and shMED12 under EGF stimulus.

**Supplemental Fig 7. ChIP-qPCR for RNAPII, INTS11, MED1 and MED12 occupancies at EGF induced genes.** HeLa cells were cross-linked with formaldehyde and harvested before and after 20 minutes of EGF-induction, with or without the presence of ERK inhibitor (SCH772984). The ChIP was performed with either IgG or antibodies against RNAPII (sc-899, Santa Cruz Biotech, *A*), INTS11 (A301-274A, Bethyl, *B*), MED1 (A300-793A, Bethyl, *C*) and MED12 (A300-774A, Bethyl, *D*). qPCR was carried out with primers at promoter region of either EGF-induced genes (NR4A1 and EGR1) or non EGF-induced genes (VAPB and NRF1). All experiments were performed in at least three biological replicates.

**Supplemental Fig 8. The deficiency of INTS11 impairs MAPK transcriptional responsiveness in cancer cells with KRAS and BRAF activating mutations.** *(A, B)* Immunoblot of KRAS-mutant lung cancer cell line A549 *(A)* and BRAF mutant melanoma cell line A375 *(B)*. The cells were treated for 3 hours with BRAF inhibitor at 1μM, MEK inhibitor at 200 nM or ERK inhibitor 1 μM of final concentration, respectively. To knockdown INTS11, the cells were transfected with siRNA (Control or INTS11 targeting) and collected at 72 hours after transfection. All the cells were undergoing 2 days of serum depletion and collected before or after 20mi EGF stimulation. *(C)* Heat maps illustrating the expression level of MAPK responsive genes (genes that were repressed concomitantly by the three inhibitors) in serum depleted A375 cells following EGF stimulation. *(D)* Dot plots showing the expression level of MAPK responsive genes (top) and control genes (bottom) in A375 cells. *(***) P* < 0.001 for corresponding comparisons.

**Supplemental Fig 9. Integrator directs MAPK transcriptional responsiveness in cancer cell lines.** *(A)* GSEA analyses reveal a positive enrichment in MAPK signaling pathways in A549 cells under EGF stimulus. *(B)* Venn diagram showing 112 EGF responsive genes in A549 cells overlap of INTS11 KD, MEK inhibitor and ERK inhibitor under EGF stimulus. *(C)* GSEA analyses reveal a negative enrichment in MAPK signaling pathways in A375 cells treated with ERK inhibitor. *(D)* Venn diagram showing 299 MAPK responsive genes found in A375 cells. Those genes that were also affected by INTS11 KD featured a reduction in gene expression by at least 40%.

**Supplemental Fig 10. The efficiency of siRNA or MAPK inhibitor treatments in cancer cells.** *(A)* Immunoblot of INTS11 under the treatment of control siRNA or siRNA against INTS11 in HeLa, A549, A375 and A375 BRAF resistant cells. *(B)* The same cells were treated with vehicle (DMSO), BRAF inhibitor (Vemurafenib, 1 µM), MEK inhibitor (PD0325901, 0.2 µM) or ERK inhibitor (SCH772984, 1 µM). Immunoblot to check phosphorylated ERK1/2, total ERK1/2 and INTS11.

**Supplemental Tables**

**Supplemental Table 1. EGF-responsive genes in HeLa cells. Related to Figure 1A and 1C, Figure 2B-D, Figure 3A and 3D.**

**Supplemental Table 2. EGF-responsive enhancers in HeLa cells. Related to Figure 1E and Figure 2E.**

**Supplemental Table 3. EGF-responsive super-enhancers (SEs) in HeLa cells. Related to Figure 1F.**

**Supplemental Table 4. Average of FPKM values of EGF-responsive genes in HeLa cells.**

**Supplemental Table 5. Serum-responsive genes in HeLa cells. Related to Figure 3F and 3H.**

**Supplemental Table 6. EGF-responsive genes in A549 cells. Related to Figure 5A and 5C.**

**Supplemental Table 7. MAPK Inhibitor responsive genes in A375 cells. Related to Figure 5D and 5F.**
